# Adolescent Girls and Young Women’s Experiences of Living with HIV in the Context of Patriarchal Culture in Sub-Saharan Africa: A Scoping Review

**DOI:** 10.1007/s10461-022-03872-6

**Published:** 2022-11-01

**Authors:** Alington Mhungu, Judith Sixsmith, Emma Burnett

**Affiliations:** School of Health Sciences, University of Dundee, 11 Arlie Place, DD1 4HN Dundee, Scotland

**Keywords:** HIV/AIDS, Adolescent girls and young women, Sub-Saharan Africa, Participation, Patriarchy

## Abstract

Adolescent girls and young women (AGYW) in sub-Saharan Africa are disproportionately affected by the human immunodeficiency virus (HIV) due to socio-cultural gender, power, and economic disparities. This scoping review examined the literature to explore what is known about AGYW’s everyday personal, relational, and social experiences of HIV to help shape future protective HIV policy and practice. Six databases were searched: Medline, CINAHL, Scopus, ASSIA, Google Scholar, and ProQuest, resulting in a total of 12,581 articles. Of these, 40 articles were included in the review. Key themes generated from the thematic analysis were relational and psychosocial challenges, inhibiting sexual expression, poverty, stigma, and discrimination; managing health in everyday life; agency and resilience; and personal space and social support. In conclusion, the review found a lack of understanding of AGYW’s everyday experiences of living with HIV from their own perspectives. There was also little consideration of the role of patriarchal culture and how this constrains AGYW’s ability to negotiate their relationships. Further research is needed to reveal AGYW’s perspectives on living with HIV in sub-Saharan Africa.

**Supplementary Information:**

The online version contains supplementary material available at 10.1007/s10461-022-03872-6.

## Introduction

Adolescent girls and young women (AGYW) are disproportionately affected by the human immunodeficiency virus (HIV). Recent statistics indicate that globally, about 5,500 young women aged 15–24 years are infected with HIV every week, and four in five new HIV infections occur in adolescent girls aged 15–19 years in sub-Saharan Africa (SSA) [1]. Key factors that negatively impact AGYW living with HIV or affected by HIV include gender inequalities, the power imbalance between men and women, and taking control of their own sexual practices [2, 3].

Research has found that some AGYW in secondary and higher education work in the sex industry to finance their education, diet, and social needs [4, 5]. AGYW often engage in transactional sexual relationships with men older than them [6], which limits their capacity to resist dominance, so they are unable to negotiate safer sex practices [7, 8]. Research also suggests that transactional sex increases AGYW’s susceptibility to sexually transmitted diseases and HIV transmission [9].

The intersectionality framework implies that AGYW’s health status may intersect with other social identities, creating interlocking systems of oppression. Scholars studying under the intersectionality framework believe that the interconnectedness of social identities such as race, class, and gender creates interdependent systems of oppression in marginalised populations [10]. An intersectionality framework is suitable for studying “*interlocking social and cultural drivers of inequity such as ethnicity, gender, age, and socioeconomic status which shape experiences of well-being*” (11, p. 4). In this context, the everyday life experiences of AGYW living with (or affected by) HIV in SSA may be understood if examined through an intersectional lens. An intersectional lens can highlight the multiple identities of those AGYW, such as the status of youth, gender, health status and positionality (living in a patriarchal culture) and how they appear to intersect, creating overlapping and interdependent systems of subordination and oppression.

Given that an estimated 300,000 HIV incidences were reported in AGYW in Eastern and Southern Africa [12] despite the availability of biomedical and socio-behavioural HIV preventive strategies [13], a gap in the current HIV response in SSA exists. To address a similar gap in the United States, the United States National AIDS Strategy recommended designing and implementing targeted and regionally tailored HIV preventive strategies [14]. Similarly, scholars scaled up research to understand the context of AGYW’s HIV experiences in SSA [13, 15–18]. Key themes identified included acceptance and sexual expression, poverty, stigma, discrimination, agency, and resilience. What was apparent was that AGYW’s everyday life and HIV experiences in SSA were influenced by patriarchal culture.

This review aims to explore available evidence and map key concepts concerning the everyday life experiences of AGYW living with (or affected by) HIV in SSA by systematically searching, selecting, and synthesising existing knowledge guided by the following research question.

What is known about the personal, relational, and social experiences of AGYW living with (or affected by) HIV/AIDS in SSA in the context of patriarchal culture?

## Methods

A scoping review was chosen as the most appropriate form of review since, according to Colquhoun et al. (2014), [19] it is a form of exploratory knowledge synthesis that addresses a broad-based review question systematically to locate and map key concepts and knowledge of a specific topic. This scoping review was guided by Arksey and O’Malley’s (2005) framework, which facilitates the comprehensive mapping and synthesising of available literature. The framework comprises the following six steps: (1) identifying a relevant review question, (2) identifying relevant studies, (3) study selection, (4) charting the data, (5) collating, summarising, and reporting results, and (6) consultation (this is an optional step) [20].

The key search terms used in the current study were: 1) **population**: “young femal*”, “young women”, “young adults”, “adolescent girls”, and “teenagers”, 2) **concept** “HIV/AIDS”, and 3) **context;** “sub-Saharan Africa”, and “patriarchy”, “patriarch*”, and “participatory research”. The Boolean operator ‘OR’ was also used, while the Boolean operator ‘AND’ was used to narrow the search results to get fewer but relevant results. Truncation (*) was applied to search for results with a root word; for example, “young femal*” was used to search for adolescent girls and young women, and quotation marks (e.g., “young female* teenagers adolescent girls and young women”) were used to combine terms instead of searching for them separately. An example of search terms and strings and their application to various databases are presented in the online supplementary Appendix I.

Six databases were searched: Medline, CINAHL, Scopus, ASSIA, Google Scholar, ProQuest, MEDLINE, and CINAHL. The Scopus database was included to increase the depth of scoping review, a technique suggested by Baas et al. (2020) [11] as it contains the largest curated citations, abstracts, and full-text articles. Grey literature was searched using the ProQuest and Google Scholar databases. The reference lists of all final selected articles were hand searched. Key websites, such as Avert global information and education on HIV and AIDS; AIDSinfo; HIV Centre for Diseases and Control Prevention (CDC); AIDS.gov; World Health Organization (WHO); and Joint UN Programme on HIV/AIDS (UNAIDS) were also searched.

As an aid to article selection, inclusion, and exclusion criteria were formulated and presented as an electronic supplement in Table 1.

The search yielded 12,578 articles as follows: Medline (2,206); CINAHL (8,724); Scopus (1,162); ASSIA (478); WHO website [2]; UNAIDS [5]; and ProQuest [1]. After removing 884 duplicates, 11,694 articles were screened by title and abstract using the inclusion and exclusion criteria in Table I. Titles and abstracts screened by AM and SA (a volunteer PhD student) excluded a total of 11,514 articles, leaving 180 full-text articles to be screened as follows. The first reviewer (AM) and the second reviewer (SA) independently screened 80% of the articles, while JS and EB screened the remaining 20%.

Two articles unavailable electronically were obtained after contacting the authors. Studies originating from and conducted in higher-income countries and an article-based study in India were excluded due to geographical settings. Further articles were excluded on the following basis: (1) two articles were excluded because they were systematic reviews and not primary studies, (2) six articles were excluded because the participants were older than the cut-off age of 24 years, and (3) three articles were excluded because they did not address AGYW experiences of HIV. A PRISMA diagram (Tricco et al., 2018) [21] was adapted and used to represent the selection procedure, and the results are presented as an electronic supplementary in Table 2.

A data charting spreadsheet adopted the Joanna Briggs Institute (JBI) manual for evidence synthesis by Peter et al. (2017) [22] to guide data extraction. The data categories used to extract data from the 40 articles selected were: author, year, and country; aims/objectives/research questions; methodology and sample; key findings and outcomes; gap and contradictions; and comments. The results of data extraction are presented as a descriptive summary and provided as an electronic supplementary in Table 3. All team members (Ali Mhungu (AM), Judith Sixsmith (JS), and Emma Burnett (EB)) were involved in this stage. To reduce selection bias, the second reviewer Salisu Abubakar independently repeated the data using the same process. The other two members of the research team, JS and EB, verified the data extraction results. Discrepancies between reviewers were resolved by discussion [23].

### Reflexive Thematic Analysis

The data were analysed and synthesised using a reflexive thematic analysis framework (RTA) to construct latent and semantic meanings. RTA is a flexible data analysis process characterised by an iterative movement back and forth between the six phases by reviewers [24]. The six phases are: familiarisation with data, coding, generating initial themes, reviewing themes, defining and naming themes, and writing up while making sense of the data. The overarching themes for this review ‘did not emerge’ from the data but were the product of a rigorous and methodical recursive and iterative constructive process [24, 25].

## Results

Of the 40 articles selected, three explored the experiences of youths, but more than half of the participants were AGYW affected by HIV/AIDS [26–28]. Five articles examined the experiences of HIV in vertically infected adolescents living with HIV [29–33], while the remaining 32 studies reported on the psychosocial experiences and impact of HIV on AGYW. As argued by Peinemann et al. (2013) [34] the results of a literature review are strengthened if they are based on an integration of different study designs. The study designs for the 40 studies included in this scoping review ranged from quantitative studies (N = 15) to retrospective cohort studies (N = 2), qualitative studies (N = 22) and mixed methods studies (N = 3). The methods used for data collection were questionnaires and surveys, face-to-face semi-structured interviews, focus group discussions and photovoice.

### Quality Assessment

Quality assessment was carried out for this scoping review, since quality assessment in scoping reviews should be considered to enhance methodological rigour [35]. In addition, quality assessment can establish whether the theoretical frameworks used were aligned to research designs and research questions, and the trustworthiness that could be attributed to the findings [36]. The studies selected for data extraction were of diverse design. Therefore, the Quality Assessment Tool Study for Diverse Designs (QATSDD) was used, as this tool is recommended for use in reviews of diverse studies because of its reliability and flexibility to assess qualitative and quantitative studies [37].

A quality assessment table is presented as an electronic supplement in Appendix 2. The QATSDD tool has 16 criteria rated on a 4-point Likert scale, ranging from 0 (not at all) to 3 (complete). All 16 items are applicable to mixed studies and 14 items are applicable to qualitative studies and quantitative studies. The total score was expressed as a percentage of the possible score. The total score per study was 42 for qualitative and quantitative designs and 48 for mixed methods, while the total score per item for the 40 studies was 120 but varied depending on whether the study was qualitative or quantitative. Included studies were assessed for quality independently by two reviewers, AM and SA (a PhD student). Disagreements were resolved either by discussion or by a third reviewer (JS). The quality of the studies was rated as follows: (0–33) % low; [34–67] % medium and [68–100] % high. The summary of the quality assessment per item demonstrated that the percentage of studies per item was variable, ranging from 38 to 76% (mean = 57.4%). Equally variable was the quality of all studies per item, ranging from 15 to 87% (mean = 62%). A low score meant that the study was less credible, while a high score indicated a more credible study.

The summary of the quality assessment per item (Appendix 2) indicated that studies scored low in two areas: use of theoretical framework 15% [18, 29–33, 38–64] and user involvement in designing studies 16% [18, 27, 29, 33, 38, 39, 41–46, 48–54, 59, 61, 63, 65–73].The studies that scored highly provided a fit between the research question and method of analysis ( quantitative only) 88%; a clear description of the procedure for data collection 86%; detailed recruitment data 86%; fit between the stated research question and method of data collection (quantitative only) 76%; fit between stated research question and format and content of data collection tool (qualitative only) 82%; a clear description of research setting 79%; and statement of aims/objective in the main body of report 74%. No studies were excluded based on poor quality assessment because scoping reviews aim not to exclude studies based on poor quality. However, quality assessment in this review enables readers to judge the findings based on methodological rigour.

There was variation in the number of participants across the methodologies. Quantitative studies ranged from 134 to 1519 participants (mean = 431.7), qualitative studies ranged from 5 to 58 (mean = 34.7), and mixed methods ranged from 40 to 539 (mean = 259.7). The distribution of studies by country is as follows: Botswana 1, Ghana 3, Kenya 4, Kenya & Uganda 2 (studies done in both countries), Malawi 3, Nigeria 2, Rwanda 1, South Africa 7, Tanzania 1, Uganda 9, and Zambia 3, and Zimbabwe 4.

Six overarching themes were developed from the reflexive thematic analysis. These were relational and psychosocial challenges, sexuality inhibition, poverty, stigma, and discrimination, managing health in everyday life, agency, resilience, personal space, and social support. The codes, sub-themes, and 6 overarching themes are presented in an electronic supplement in Appendix 3.

### Relational and Psychosocial Challenges

This theme relates to the relational and psychosocial challenges faced by AGYW living with (or affected by) HIV, which impact their everyday lives. Twenty studies reported relational and psychosocial challenges [28, 29, 31, 45, 49, 51, 53, 55–57, 60–62, 64, 69, 72, 74–77]. The findings indicated that relational factors affecting AGYW included failure to build interpersonal relationships due to fear of rejection and isolation [28, 33, 45, 53, 57]. For example, AGYW experienced challenges in developing healthy relationships after the loss of parent(s) because they continuously relocated to live with different relatives [78]. Constant relocation meant that AGYW experienced isolation and rejection and struggled to adjust to new families and make new friends due to the HIV-related stigma [31, 49, 51, 55].

The psychosocial challenges experienced included accepting the HIV diagnosis, taking antiretroviral therapy for life, and attending regular hospital appointments. The impact of these psychosocial challenges included episodes of depressive disorder and suicidal ideation [29, 49, 51, 59, 76]. Similarly, the presence of psychological distress causing dysfunctional behaviour was also reported [45, 51, 61, 65, 79, 80]. Experiences of dysfunctional behaviours and depressive disorders are particularly problematic because they are often associated with excessive drug and alcohol use and a tendency to engage in risky sexual behaviour [81, 82]. In addition, intoxication reduces adherence to high active antiretroviral therapy (HAART) [83] known to increase HIV viral load while engaging in risky sexual risk behaviours. For example, intergenerational sex is a risk factor for onward HIV transmission [6].

### Inhibiting Sexual Expression

Nine studies described how AGYW living with HIV were inhibited from expressing their sexuality fully because of misconceptions about their HIV identity and lack of availability of sexual and reproductive health services [33, 38, 39, 44, 47, 49, 53, 63, 73]. Additionally, AGYW and their communities appear to be unaware that HIV cannot be transmitted if people with HIV take HAART consistently and have an undetectable viral load [44, 47]. As a result of their lack of knowledge, AGYW were fearful of transmitting HIV to their children.

Inaccessible sexual and reproductive health (SRH) services were associated with AGYW’s failure to insist on safe sex methods such as using condoms [44, 49, 73]. For example, [38] found that AGYW who did not access SRH and contraceptive counselling reported unwanted pregnancies. Similarly, some AGYW experienced constrained sexual partnerships [33, 39, 53, 73]. Constrained sexual relationships were experienced when AGYW knew that they should disclose their HIV status to their sexual partners. Yet, the fear of rejection and HIV-associated stigma prevented them not only from disclosing their HIV status but also from exploring their sexuality fully. In the same context, statistics in Malawi showed that adolescent pregnancy rates were 141/1000 girls, known to be three-fold the global average [84]. This high teenage pregnancy rate appears to be associated with the poor availability of SRH services. Accessing sexual and reproductive health is essential, as it can reduce child marriage, teenage pregnancies, and transmission of sexually transmitted diseases, including HIV.

### Poverty, Stigma, and Discrimination

AGYW living with (or affected by) HIV were found to be affected by poverty, stigma, and discrimination [33, 38, 43, 44, 46, 48, 49, 52, 55, 59, 73, 85]. Three studies found that poverty was a distinct hindrance to AGYW’s health and well-being because poverty acted as a barrier to managing health in their everyday life [43, 46, 52]. For example, poor AGYW struggled to raise cash for basic needs such as school fees, bus fares to attend HIV clinic appointments, and food [43, 46, 52]. The lack of food is problematic for those taking HAART, as medication must be taken with food so as not to irritate the stomach lining [86]. Some studies have reported that individuals often miss taking HAART because of the lack of availability of food [87, 88]. Similarly, food insecurity was consistently found to be associated with higher rates of sexual risk behaviour, intimate transactional sexual relationships and barriers to HAART initiation/adherence [89].

This review found that AGYW continue to experience enacted stigma and felt discrimination within their social context [38, 43, 44, 48, 49, 53, 55, 59]. Enacted stigma is external stigma from the public based on the condition society perceives to be imperfect [90]. For example, an adolescent girl cited how she experienced stigma in the community: “*My teacher saw me at the ART clinic and later disclosed to my classmates that I am HIV-positive and on drugs. I tried to drown in a pond, but it was too shallow”* (15, p. 11). Thus, AGYW’s everyday life experiences appeared to be characterised by pain, fear, and a sense of injustice, particularly since they were stigmatised despite acquiring HIV at birth. This claim is confirmed by [18], whose study found that adolescents living with HIV attending school lived in fear of stigma and unintentional disclosure.

### Managing Health in Everyday life

This theme was widely reported, as indicated by the following studies [30, 32, 33, 38, 45, 47, 52, 55, 57, 60, 61, 65, 74, 85, 91–93]. Managing health in everyday life describes how AGYW living with HIV overcome the many challenges they face and find ways to adapt and live with the HIV diagnosis. Nine studies reported that HIV-positive AGYW used ‘social comparison’ to come to terms with their HIV diagnosis [28, 30, 31, 41, 44, 49, 51, 64, 73]. For example, social comparison is often used by people with newly diagnosed chronic conditions to adjust to new illnesses and manage their health [94]. In this context, AGYW compared themselves with other AGYW living with or affected by HIV. Knowing that other AGYW were living with HIV enabled participants to accept their HIV diagnosis [33, 38, 59]. Research suggests that pairing people with a newly diagnosed condition with someone coping well or with a more severe illness can produce positive health outcomes [91].

Emotionally connecting with peers at the HAART clinics was another way in which AGYW managed their health and well-being [33]. The environment at HAART clinics provided AGYW with a safe space to meet peers and discuss HIV health issues without fear of unintended disclosure and stigma. This review suggests that AGYW were motivated to maintain HAART adherence because it eliminated stigmatising physical symptoms of HIV infection, such as severe loss of weight known to be connected to HIV [33, 38, 55, 57, 59].

### Agency and Resilience

Several studies proposed that AGYW used agency and resilience to overcome the adversity of living with HIV, such as HIV-related stigma and discrimination and lack of social support [26, 28, 30, 31, 33, 38, 44, 47, 51–53, 55, 92, 95]. Agency in this context relates to the capacity for AGYW to participate in decision making concerning their health and issues affecting their everyday life. AGYW demonstrated agency and adopted strategies of secrecy and silence to manage their HIV identities [95]. For example, some AGYW maintained their choice to decide when and to whom they disclosed their HIV status. Contrary to discourses of passivity and victimisation in marginalised women [96], AGYW living with HIV also articulated their preferences for community peer mentors to healthcare workers because peers had experienced HIV, HIV-related stigma and being on HAART [30, 52]. One participant in a related study demonstrated self-determination to cope with the pain of losing her parents to HIV by going to the graveyard every Sunday to ‘speak’ to her deceased parents [54]. Despite her guardian preferring to deal with grief by praying, the young girl showed agency by designing her own strategy to cope with her grief.

This review found that AGYW showed resilience in their everyday lives. In contrast, previous research on women/AGYW and HIV has focused on the challenges they face while neglecting to explore their strengths or resilience [97]. Resilience has two elements: (i) exposure to adversity and (ii) positive adaptation despite experiencing adversity [98]. In this context, AGYW living with (or affected by) HIV adapted and successfully lived everyday lives and planned for a successful future. Resilience was demonstrated by a participant who said, *“I like school and want to be a journalist to warn other girls to desist from boys… I will collect information from HIV-positive girls and write it in the newspaper”* (85, p. 6). Similarly, AGYW affected by HIV demonstrated altruism and coped with the burden of caring for terminally ill HIV relatives by drawing inspiration from the positive cultural roles of helping their parents [57, 59]. AGYW drew inspiration from their culture, which valued education and caring for the infirm. These AGYW developed positive goals and demonstrated motivation to succeed in life despite the adversity of living with HIV.

Resilience is also dependent on self-efficacy. Self-efficacy is described as people’s belief that they can succeed in a given task [99]. Self-efficacy may be influencing breastfeeding behaviour for AGYW in SSA experiencing the intersections of hunger, poverty, HIV status and cultural norms known to increase the vulnerability of mother-to-child HIV transmission [100]. For example, young mothers persistently walk a long distance to access HIV care and HAART to keep their infants free of HIV [52]. According to the WHO guidelines on HIV and infant feeding, consistent medication with HAART and breastfeeding are critical for preventing the death of children aged less than five years and preventing mother-to-child HIV infection [101].

### Personal Space and Social Support

This theme examined the physical environment’s integral role and the community’s social support in the experiences of AGYW living with (or affected by) HIV. Studies found that the lack of personal and safe space to take HAART in schools and social support affected the health and well-being of AGYW [26, 29, 43, 44, 46, 60, 64]. For example, AGYW experienced a lack of personal space in schools to take HAART missed doses [18, 46]. This finding is supported by [102], who found that HAART adherence was associated with privacy and personal space availability. In addition, a lack of safe space, particularly in boarding schools, might have influenced students to change schools after unintended HIV disclosure and related stigma [18]. HAART adherence is essential because the inconsistent taking of HAART can increase HIV viral load, vulnerability to opportunistic infections, and the risk of onward HIV infection.

AGYW’s everyday life experiences were found to be influenced by social support in the community in this review. For instance, a 16-year-old girl living with HIV illustrated how lack of social support could be viewed as subtle HIV-related discrimination at home: *“I was made to do all the house chores at home while other children went to school”* (15, p. 11). Similarly, the existence of perceived social support disparities among children affected by HIV/AIDS [103] influenced their HIV experiences. The unjustified discrimination of AGYW in the community relates to entrenched traditional beliefs in SSA associating HIV infection with immorality [104]. As a result, some AGYW are stigmatised, yet they may have acquired HIV at birth [105]. However, the review also found that AGYW’s health and well-being were enhanced by social support from trusted family members, friends, support groups and interacting health workers and peer educators [73]. Similarly, AGYW living with HIV provided social support for peers in their communities through support group networks [62]. In a related study, emotional support from the family, schoolteachers, classmates, and friends was found to be associated with better grades and well-being of adolescents living with HIV [106].

## Discussion

The review set out to explore the everyday life experiences of AGYW living with (or affected by) HIV/AIDS in SSA in the context of patriarchal culture. This review aim is discussed in relation to key findings, the date of articles, and the countries where the studies were located.

This review found that some AGYW living with HIV struggled with depression and building interrelationships that negatively impacted their mental health (50,51,107,). This finding is consistent with previous studies that reported that HIV orphans experienced anxiety, post-traumatic disorder, and misconduct, and struggled to build relationships with their peers [108, 109]. AGYW’s failure to develop close relationships is also related to how women with severe chronic heart failure felt isolated because of a lack of social interactions [110]. Similarly, a lack of social support socially isolated AGYW, making them susceptible to mental health illness [107]. This finding is consistent with recent research that found that mental health problems and lack of sexual and reproductive health services increase the risk of onward HIV infections [107, 111]. AGYW who lack access to sexual and reproductive services are likely to engage in sexual risk behaviours, have multiple sex partners and have poor condom compliance that may lead to pregnancy and sexually transmitted diseases, including HIV [112, 113]. HIV prevalence continues to be associated with low sexual and reproductive health (SRH) in SSA, according to Melesse et al. (2020) [114]; this is caused by major differences in SRH services between regions, countries, and within countries. To lower HIV prevalence, there is a need for country-specific research and to develop age- and country-specific sexual and reproductive strategies.

Failure to express sexuality was a key finding of this review. This appeared to be associated with an inability to access sexual and reproductive health services. Some AGYW identified a lack of access to sex education and contraceptive counselling, which subsequently affected their ability to discuss their sexuality and birth control [49]. One of the limitations of the previous wider literature on sexual and reproductive health is that it focuses on the challenges and barriers to access, neglecting how AGYW construct their sexuality in the context of HIV. Data on how AGYW construct sexuality is essential to develop tailored HIV strategies for AGYW to prevent HIV infection and onward transmission [115]. Expressing sexuality is important for AGYW in SSA, where intergenerational sexual relationships, gender-based violence, and HIV stigma intersect under the dominant patriarchal culture to further marginalise AGYW [9]. Given that sexual and reproductive strategies for women living with HIV in SSA are not supported well by national policy [116], the WHO designed a theory of change for sexual and reproductive health and HIV linkages [117]. This theory comprises a more integrated HIV and sexual and reproductive health service delivery to reduce gender-based violence and HIV stigma. This needs to be implemented across SSA to improve health and human rights and reduce the prevalence of HIV.

There is evidence that sexual and reproductive health prepares and empowers AGYW to manage gender inequalities and resist sexual coercion and exploitation [118, 119]. However, this review found that a lack of accurate sexual and reproductive knowledge inhibited AGYW from expressing their sexuality. This finding is critical because the data can be used to develop future sexual and reproductive health services and practices for AGYW in SSA. It might be essential to include sexual and reproductive health in comprehensive HIV care for AGYW to improve their health and well-being. Sexual and reproductive health might empower AGYW to resist intergenerational sex relationships known to expose HIV-positive AGYW to sexually transmitted diseases and the risk of onward HIV transmission [107, 120]. In addition, well-informed AGYW living with HIV can engage in safe sex practices and adhere to HAART, which prevents mother-to-child HIV transmission.

This review identified that AGYW experience HIV-related stigma and discrimination. Stigma is defined as “an attribute that is deeply discrediting that devalues a person from a whole to a tainted and discounted one” (121, p. 3). HIV stigma occurs at interpersonal, social, and individual levels, affecting people perceived to have a tainted and discrediting attribute in their social interactions [122]. This is consistent with a previous study by Pryor and Reeder (2011) [123] suggesting that a person perceived to have a discrediting attribute may experience psychological and social sanctions in their everyday lives. Despite antiretrovirals changing HIV from a terminal to a chronic disease, HIV stigma and discrimination at home and in the community continue to be difficult challenges for people living with HIV/AIDS [124]. Stigmatising people living with HIV has a historical background, since contracting HIV is considered a punishment for bad behaviour in traditional communities in SSA [125–127].

A history of HIV stigma is associated with the perception of the norm violation model [128, 129]. Norm violation refers to collective norms or values of what is acceptable or not acceptable behaviour, with sanctions enforced to shape community members’ behaviour. Historically, the norm violation model is dominant in patriarchal societies, perpetuating the belief that certain illnesses are caused by deviant behaviours [127]. The perception that HIV is contracted through promiscuity, homosexuality, and drug abuse is the reason why conservative communities react with anger and the social ostracisation of people living with or affected by HIV in SSA. In addition, religious beliefs in SSA attach an immoral label to people living with HIV because HIV is perceived to be a punishment for bad behaviour [104]. Consistent with the intersectionality perspective [130], HIV health status, norm violation and gender disparities intersected, creating felt stigma and further marginalising AGYW in their everyday life experiences.

This review also found that AGYW experienced stigma related to a lack of personal space to take HAART in private in schools and higher education [43, 60]. Lack of personal space in boarding schools heightened fear of unintended HIV disclosure and stigma because students were not prepared to take HAART in the presence of classmates [18]. AGYW’s fear of HIV disclosure and the desire to maintain silence and secrecy need to be conceptualised in the prevailing cultural and social construction of stigmatised illnesses [131]. Research suggests that HIV remains highly stigmatised, and unintended disclosure can lead to exclusion from school, forced relocations and loss of employment in Indonesia; hence, “Silence is the preferred and most liveable strategy for most people living with HIV” (132, p. 3). Similarly, a student in Namibia captured how the intersection of social stigma and HIV health status shaped a strategy for silence and secrecy. She said, “*Another problem with the tablets is that I have to go to the toilet or somewhere where there are no other learners to take them. I know that if they see me taking these, they will tease me”* (133, p. 241). This finding has implications for future policy and practice. The implication for practice is that educational facilities must accommodate students living with HIV by creating personal and safe spaces. The rationale for such a strategy is that a lack of personal and secure space may result in missed HAART doses and perpetuate the shame, secrecy, and stigma associated with HIV [18].

Felt stigma is described as knowledge and anticipation that a person will be discriminated against based on an attribute that is socially deemed undesirable [90]. For AGYW living with HIV, felt stigma triggered feelings of guilt, shame, worthlessness, and subsequent depression at a personal level [134]. One participant in this review opted to commit ‘slow suicide’ by not taking HAART because she anticipated being stigmatised due to the HIV diagnosis [46]. Given that HIV-related stigma occurs at the individual, interpersonal, and social levels, interventions to reduce it may be more effective if they target all these levels.

Surprisingly, this review found that living with (or being affected by) HIV did not stop some AGYW from living a healthy life [45, 50, 85]. Instead, AGYW appeared to use their agency and resilience to adapt to and manage health in their everyday lives. The feminist theory of autonomy and agency can be drawn upon to interpret this finding. The theory posits that agency is influenced by self-definition. Self-definition occurs when people reflect and become aware of themselves and conceptualise themselves as socially and culturally constituted [135]. The implication for AGYW is that by reflecting and understanding that social and cultural norms shape their oppressions, opportunities, and vulnerabilities, they might be empowered to recognise and resist disabling norms that constrain them [136]. Given that this review found that AGYW used agency to guide their decision making, future research is recommended to focus on investigating how AGYW living with (or affected by) HIV use agency to protect their health. Including the voice of young women in developing everyday practices and strategies they use in their daily lives alongside policies to promote their agency will also help formulate interventions that will empower this marginalised group.

This review found that AGYW are inhibited from expressing their sexuality in SSA [33, 38, 39, 44, 47, 49, 53, 73, 137]. Previous research found that women are at the highest risk of poor health outcomes associated with the intersecting oppressive structural systems because their choices and ability to exercise power are stifled by the patriarchal culture in which they live [138, 139]. Similarly, the health status of AGYW and the deeply entrenched traditional beliefs in SSA are intricately interwoven and impact their everyday life experiences. To improve the health outcomes of AGYW, to enable health choices around sexual relationships and reduce HIV burden, it is important to understand the situations and circumstances of AGYW in their attempt to negotiate sex in their everyday life in personal, relational, and social contexts. Such experiential knowledge can help craft HIV policy and practice that resonates with their situations, is relevant to their lives and therefore more likely to work in preventive ways. However, little is known about how AGYW living with (or affected by) HIV act to manage the gender disparities they experience in their everyday lives that link to HIV infection, or what strategies they use to protect health and well-being within sexual relationships.

This strongly points towards the need for more participatory ways of working with AGYW to enable their voices to be heard and acted upon. The principle of participation in healthcare has grown over recent years [140]. Patient and public involvement enable marginalised populations like AGYW to be involved in decisions that affect their health, including defining the problem, planning, implementing, and evaluating interventions [141]. Similarly, Grocott et al. [142] found that while patients’ key priority is good management or recovery from ill health, they also want to be involved in shared decision making regarding their healthcare. Given that this review indicated the importance of understanding the impact of patriarchal norms on AGYW’s everyday life experiences of HIV in SSA, future studies are needed to explore AGYW’s experiential knowledge of HIV in SSA in terms of their personal, relational, and social contexts. It is hoped that enabling AGYW to participate in decisions about their health might effectively empower them to address structural and intersecting pre-existing vulnerabilities like poverty, gender disparities, and youth and HIV health status [143]. The generated knowledge from such research can also be used to identify the needs of AGYW and develop culturally sensitive strategies to promote gender equality, their health, and well-being.

The literature on AGYW’s experiences of living with HIV is limited because it was informed by research from 11 out of 46 SSA countries. These 11 countries are in Eastern and Southern Africa, Central Africa, and West Africa [144]. Therefore, it can be argued that this knowledge may not be transferable to other HIV hotspot countries in SSA, where no research has been carried out. eSwatini and Swaziland (two countries with the first and second highest global HIV prevalence) [144] have unique historical characteristics that may affect the experiences of AGYW living with HIV. These two countries extensively practise patriarchal norms, including polygamy, and most men migrate to work in South African mines, both known risk factors for HIV transmission [145].

This review found that AGYW used resilience to cope with the everyday life challenges of living with HIV. Resilience is a process describing how people adapt to overcome experiences of stress following trauma or the diagnosis of chronic illness [146]. Resilience in marginalised women can be unpacked using two conceptual frameworks: photovoice and feminist critical consciousness [136]. There is congruence between these two frameworks. Participants can use experiential knowledge and dialogue to critically reflect on their strengths and constraints to initiate social change and improve their everyday lives [136, 147]. The importance of this approach is that it empowers marginalised people to identify the problems affecting them and co-design solutions. In the same context, this review found that AGYW living with HIV who preferred to work with peers as mentors instead of healthcare workers [30, 52], created synergies facilitated by community adolescent treatment supporters to maintain autonomy in their lives [51]. Given that AGYW used resilience as a critical strategy to cope with living with HIV, future research studies may adopt photovoice design supported by an intersectional framework to understand how HIV status intersects with social identities, creating unique systems of oppression for AGYW. This type of research design is likely to facilitate understanding AGYW’s experiences of HIV from their perspectives, enabling their voices to be heard.

This scoping review has several strengths. First, the review included articles with diverse study designs, such as qualitative (N = 22), quantitative (N = 15), and mixed methods (N = 3), thereby improving the breadth and depth of the findings. Second, this review adopted a structured methodology for identifying the review questions, extensive literature search, selecting studies, charting, and mapping data, and collating and reporting data [20, 22, 35]. Third, the need for AGYW’s experiential knowledge necessary for crafting HIV policy and practice sensitive to and therefore more relevant to AGYW’s needs was highlighted. Fourth, a team of reviewers (AM, SA, JS, and EB) independently screened and selected data with disagreements resolved through discussion or by a third reviewer.

Four key limitations were identified.1) The studies included in this review were limited to SSA. Therefore, generalising the findings to other regions should be approached with caution. 2) The scoping review covers a range of countries; the findings may not be relevant to all countries. 3) The literature search was limited to studies published in English. Failure to include non-English studies means that studies published in other languages were excluded [148], so further research in this area is advised. 4) Qualitative research does not routinely engage in quality assurance. Given that qualitative research is informing global health programmes, it has become increasingly essential that qualitative research passes external scrutiny and demonstrates confidence in the interpretation of results [149]. Therefore, a quality assessment was undertaken to demonstrate the methodological rigour of the studies included in this scoping review. In addition, a quality assessment might also enable readers to better understand the extent of the trustworthiness of the findings.

The quality assessment indicated that the studies included in this review scored low only in two items: explicit theoretical framework and evidence of user involvement in design. However, methodological rigour was enhanced because all the studies scored medium to high on 14 out of 16 items on the Quality Assessment Tool Study for Diverse Designs.

## Conclusion

This scoping review found that AGYW living with HIV in SSA experienced relational and psychosocial challenges. Some AGYW experienced anxiety and post-traumatic disorder, which appeared to have an impact on the development of relationships with their peers. In addition, psychosocial distress affected AGYW’s interpersonal relationships, where fear of rejection and stigma delayed HIV status disclosure to key family members and partners. Failure to disclose AGYW’s HIV status to significant others is detrimental to them and their sex partners in the wider society. This review identified a research gap; therefore, further research is recommended.

## Electronic Supplementary Material

Below is the link to the electronic supplementary material.

Supplementary Material 1

Supplementary Material 2

Supplementary Material 3

Supplementary Material 4

Supplementary Material 5

Supplementary Material 6

## Data Availability

Not applicable.

## References

[1] Joint United Nations Programme on HIV and AIDS. Global HIV Statistics [Internet]. UNAIDS. 2020. Available from: https://www.unaids.org/en/resources/fact-sheet.

[2] Namy S, Carlson C, O’Hara K, Nakuti J, Bukuluki P, Lwanyaaga J, et al. Towards a feminist understanding of intersecting violence against women and children in the family. *Soc Sci Med* [Internet]. 2017;184:40–8. Available from: doi:10.1016/j.socscimed.2017.04.042. 10.1016/j.socscimed.2017.04.042 PMC5737762 28501019

[3] Amnesty international. “Lost without knowledge”: Barriers to sexual and reproductive healthcare information in Zimbabwe [Internet]. 2018 [cited 2019 Jan 28]. Retrieved from www.amnesty.org.

[4] Ranganathan M, MacPhail C, Pettifor A, Kahn K, Khoza N, Twine R, et al. Young women’s perceptions of transactional sex and sexual agency: A qualitative study in the context of rural South Africa. *BMC Public Health* [Internet]. 2017 Dec 22 [cited 2018 Nov 27];17(666):1–16. Available from: 10.1186/s12889-017-4636-6. 10.1186/s12889-017-4636-6 PMC5568133 28830394

[5] Mitiku Dana L, Mehretie Adinew Y, Molla Sisay M, Dana LM, Adinew YM, Sisay MM (2019). Transactional sex and HIV risk among adolescent school girls in Ethiopia: Mixed method study. Biomed Res Int.

[6] Schaefer R, Gregson S, Eaton JW, Mugurungi O, Rhead R, Takaruza A, et al. Age-disparate relationships and HIV incidence in adolescent girls and young women: Evidence from Zimbabwe. AIDS [Internet]. 2017 Jun 19 [cited 2019 Feb 12];31(10):1461–70. Available from: doi:10.1097/QAD.0000000000001506. 10.1097/QAD.0000000000001506 PMC5457819 28426534

[7] Kishor S. Intimate partner violence and HIV: Clearing up confusion [Internet]. Vol. 3, *The Lancet Global Health*. 2015 [cited 2018 Nov 13]. Available from: http://www.thelancet.com/lancetgh. 10.1016/S2214-109X(14)70372-9 25539968

[8] Beauclair R, Dushoff J, Delva W. Partner age differences and associated sexual risk behaviours among adolescent girls and young women in a cash transfer programme for schooling in Malawi. *BMC Public Health* [Internet]. 2018 Mar 27 [cited 2019 Jun 25];18(1):1–12. Available from: 10.1186/s12889-018-5327-7. 10.1186/s12889-018-5327-7 PMC5872581 29587710

[9] Wamoyi J, Stobeanau K, Bobrova N, Abramsky T, Watts C. Transactional sex and risk for HIV infection in sub-Saharan Africa: A systematic review and meta-analysis. *J Int AIDS Soc* [Internet]. 2016 [cited 2019 Jul 17];19(20992):1–16. Available from: 10.7448/IAS.19.1.20992. 10.7448/IAS.19.1.20992 PMC5095351 27809960

[10] Algarin AB, Zhou Z, Cook CL, Cook RL, Ibañez GE. Age, Sex, Race, Ethnicity, Sexual Orientation: Intersectionality of Marginalized-Group Identities and Enacted HIV-Related Stigma among People Living with HIV in Florida. *AIDS Behav* [Internet]. 2019;23(11):2992–3001. Available from: 10.1007/s10461-019-02629-y. 10.1007/s10461-019-02629-y PMC6803104 31392442

[11] Baas J, Schotten M, Plume A, Côté G, Karimi R (2020). Scopus as a curated, high-quality bibliometric data source for academic research in quantitative science studies. Quant Sci Stud.

[12] UNICEF. Children. and AIDS: Statistical update [Internet]. www.Childrenandaids.Org.2017. Available from: https://data.unicef.org/wp-content/uploads/2017/11/HIVAIDS-Statistical-Update-2017.pdf.

[13] Zuma T, Seeley J, Mdluli S, Chimbindi N, Mcgrath N, Floyd S, et al. Young people’s experiences of sexual and reproductive health interventions in rural KwaZulu-Natal, South Africa. *Int J Adolesc Youth* [Internet]. 2020;25(1):1058–75. Available from: 10.1080/02673843.2020.1831558. 10.1080/02673843.2020.1831558 PMC8224946 34177039

[14] Abrams JA, Odlum M, Tillett E, Haley D, Justman J, Hodder S, et al. Strategies for increasing impact, engagement, and accessibility in HIV prevention programs: Suggestions from women in urban high HIV burden counties in the eastern United States (HPTN 064). *BMC Public Health* [Internet]. 2020;20(1):1–16. Available from: 10.1186/s12889-020-09426-6. 10.1186/s12889-020-09426-6 PMC7469400 32883248

[15] Kimera E, Vindevogel S, Reynaert D, Justice KM, Rubaihayo J, de Maeyer J, et al. Experiences and effects of HIV-related stigma among youth living with HIV/AIDS in Western Uganda: A photovoice study. *PLoS One* [Internet]. 2020;15(4):1–21. Available from: 10.1371/journal.pone.0232359. 10.1371/journal.pone.0232359 PMC7182188 32330206

[16] Mccarraher DR, Packer C, Mercer S, Dennis A, Banda H, Nyambe N, et al. Adolescents living with HIV in the Copperbelt Province of Zambia: Their reproductive health needs and experiences. *PLoS One* [Internet]. 2018;13(6):1–13. Available from: 10.1371/journal.pone.019785. 10.1371/journal.pone.0197853 PMC5988282 29870562

[17] Kaunda-Khangamwa BN, Kaunda-Khangamwa BN, Kaunda-Khangamwa BN, Kapwata P, Malisita K, Munthali A, et al. Adolescents living with HIV, complex needs and resilience in Blantyre, Malawi. *AIDS Res Ther* [Internet]. 2020;17(1):1–13. Available from: 10.1186/s12981-020-00292-1. 10.1186/s12981-020-00292-1 PMC7310029 32571375

[18] Madiba S, Josiah U. Perceived stigma and fear of unintended disclosure are barriers in medication adherence in adolescents with perinatal HIV in Botswana: A qualitative study. *Biomed Res Int* [Internet]. 2019 [cited 2021 Aug 21];2019:1–10. Available from: 10.1155/2019/9623159. 10.1155/2019/9623159 PMC6914939 31886271

[19] Colquhoun HL, Levac D, O’Brien KK, Straus S, Tricco AC, Perrier L, et al. Scoping reviews: Time for clarity in definition, methods, and reporting. *J Clin Epidemiol* [Internet]. 2014 [cited 2019 Aug 8];67:1291–4. Available from: 10.1016/j.jclinepi.2014.03.013. 10.1016/j.jclinepi.2014.03.013 25034198

[20] Arksey H, O’Malley L. Scoping studies: Towards a methodological framework. *Int J Soc Res Methodol* [Internet]. 2005 Feb [cited 2019 Jul 31];8(1):19–32. Available from: http://www.tandfonline.com/doi/abs/10.1080/1364557032000119616.

[21] Tricco AC, Lillie E, Zarin W, Brien KKO, Colquhoun H, Levac D, et al. PRISMA Extension for Scoping Reviews (PRISMA-ScR): Checklist and Explanation. *Ann Intern Med* [Internet]. 2018;169(7):467–73. Available from: doi: 10.7326/M18-0850. 10.7326/M18-0850 30178033

[22] Peters M, Godfrey CM, Mcinerney P, Baldini Soares C, Khalil H, Parker D. Chapter 11: Scoping Reviews: 2017 Guidance for the Conduct of JBI Scoping Reviews [Internet]. Aromatis, E. Munn. Z, editor. Vol. 13, *Joanna Briggs Institute Reviewer’s Manual*. The Joanna Briggs Institute. 2017. 141–146 p. Available from: https://reviewersmanual.joannabriggs.org/display/MANUAL/Chapter+11%3A+Scoping+reviews The.

[23] Waffenschmidt S, Knelangen M, Sieben W, Bühn S, Pieper D. Single screening versus conventional double screening for study selection in systematic reviews: A methodological systematic review. *BMC Med Res Methodol* [Internet]. 2019 [cited 2021 Aug 21];19(132):1–9. Available from: 10.1186/s12874-019-0782-0. 10.1186/s12874-019-0782-0 PMC6599339 31253092

[24] Braun V, Clarke V. Reflecting on reflexive thematic analysis. *Qual Res Sport Exerc Heal* [Internet]. 2019 [cited 2020 Jan 31];11(4):589–97. Available from: https://www.tandfonline.com/action/journalInformation?journalCode=rqrs21.

[25] Braun V, Clarke V. Using thematic analysis in psychology. *Qual Res Psychol* [Internet]. 2006 [cited 2020 Jan 9];3(2):77–101. Available from: http://www.tandfonline.com/loi/uqrp20.

[26] Skovdal M, Ogutu VO. “I washed and fed my mother before going to school”: Understanding the psychosocial well-being of children providing chronic care for adults affected by HIV/AIDS in Western Kenya. Global Health [Internet]. 2009 [cited 2019 Nov 10];5(8):1–8. Available from: doi = 10.1186%2F1744-8603-5-8. 10.1186/1744-8603-5-8 PMC2736916 19698177

[27] Demmer C. Experiences of families caring for an HIV-infected child in KwaZulu-Natal, South Africa: An exploratory study. *AIDS Care* [Internet]. 2011; 23(7):873–9. Available from: doi.10.1080/09540121.2010.542123. 10.1080/09540121.2010.542123 PMC3125458 21400305

[28] Thupayagale-Tshweneagae G. Behaviours used by HIV-positive adolescents to prevent stigmatization in Botswana. *Int Nurs Rev* [Internet]. 2010 Jun [cited 2019 Nov 11];57(2):260–4. Available from: http://doi.wiley.com/10.1111/j.1466-7657.2009.00792.x. 10.1111/j.1466-7657.2009.00792.x 20579163

[29] Mutwa PR, Ilo J, Nuil V, Asiimwe-Kateera B, Kestelyn E, Vyankandondera J (2013). Living situation affects adherence to combination antiretroviral therapy in HIV-infected adolescents in Rwanda: A qualitative study. PLoS One [Internet].

[30] Kidia KK, Mupambireyi Z, Cluver L, Ndhlovu CE, Borok M, Ferrand RA (2014). HIV status disclosure to perinatally-infected adolescents in Zimbabwe: A qualitative study of adolescent and healthcare worker perspectives. PLoS One [Internet].

[31] Mutumba M, Musiime V, Tsai AC, Byaruhanga J, Kiweewa F, Bauermeister JA, et al. Disclosure of HIV status to perinatally infected adolescents in urban Uganda: A qualitative study on timing, process, and outcomes. *JANAC J Assoc Nurses AIDS Care* [Internet]. 2015;26(4):472–84. Available from: doi: 10.1016/j.jana.2015.02.001. 10.1016/j.jana.2015.02.001 26066697

[32] Nkwata AK, Zalwango SK, Kizza FN, Sekandi JN, Mutanga J, Zhang M, et al. Quality of life among perinatally HIV-affected and HIV-unaffected school-aged and adolescent Ugandan children: A multi-dimensional assessment of wellbeing in the post-HAART era. *Qual Life Res* [Internet]. 2017 Sep;26(9):2397–408. Available from: doi.10.1007/s11136-017-1597-2. 10.1007/s11136-017-1597-2 28534093

[33] Enimil A, Nugent N, Amoah C, Norman B, Antwi S, Ocran J, et al. Quality of life among Ghanaian adolescents living with perinatally acquired HIV: A mixed methods study. *AIDS Care - Psychol Socio-Medical Asp AIDS/HIV* [Internet]. 2015 [cited 2019 Nov 12];28(4):460–4. Available from: 10.1080/09540121.2015.1114997. 10.1080/09540121.2015.1114997 PMC4764441 26643735

[34] Peinemann F, Tushabe DA, Kleijnen J (2013). Using multiple types of studies in systematic reviews of health care interventions: A systematic review. PLoS One [Internet].

[35] Levac D, Colquhoun H, O’brien KK. Scoping studies: Advancing the methodology. *Implement Sci* [Internet]. 2010 [cited 2019 Aug 8];5(69):1–9. Available from: http://www.implementationscience.com/content/5/1/69%0APage. 10.1186/1748-5908-5-69 PMC2954944 20854677

[36] *Clin Teach* [Internet]. 2020;17(6):596–9. Available from: doi: 10.1111/tct.13242. 10.1111/tct.13242 32790137

[37] Sirriyeh R, Lawton R, Gardner P, Armitage G. Reviewing studies with diverse designs: The development and evaluation of a new tool. *J Eval Clin Pract* [Internet]. 2012;18(4):746–52. Available from: doi: 10.1111/j.1365-2753.2011.01662.x. 10.1111/j.1365-2753.2011.01662.x 21410846

[38] Zamudio-Haas S, Mudekunye-Mahaka I, Lambdin BH, Dunbar MS. If, when, and how to tell: A qualitative study of HIV disclosure among young women in Zimbabwe. *Reprod Health Matters and Int J Sex Reprod Health Care* [Internet]. 2012 [cited 2019 Nov 12];12(39S):18–26. Available from: 10.1016/S0968-8080(12)39637-7. 10.1016/S0968-8080(12)39637-7 23177677

[39] Busza J, Vr Besana G, Mapunda P, Oliveras E. “I have grown up controlling myself a lot.” Fear and misconceptions about sex among adolescents vertically infected with HIV in Tanzania. *Reprod Health Matters* [Internet]. 2013 [cited 2019 Nov 11];21(41):87–96. Available from: 10.1016/S0968-8080(13)41689-0?. 10.1016/S0968-8080(13)41689-0 23684191

[40] Lowenthal ED, Marukutira TC, Chapman J, Mokete K, Riva K, Tshume O, et al. Psychosocial assessments for HIV + African adolescents: Establishing construct validity and exploring under-appreciated correlates of adherence. *PLoS One* [Internet]. 2014 [cited 2019 Nov 11];9(10):1–8. Available from: http://www.med.upenn.edu/botswana/. 10.1371/journal.pone.0109302 PMC4184864 25279938

[41] Mburu G, Hodgson I, Kalibala S, Haamujompa C, Cataldo F, Lowenthal ED, et al. Adolescent HIV disclosure in Zambia: Barriers, facilitators, and outcomes. *J Int AIDS Soc* [Internet]. 2014 [cited 2021 Aug 22];17(1):1–9. Available from: 10.7448/IAS.17.1.18866. 10.7448/IAS.17.1.18866 PMC3956312 24629845

[42] Nöstlinger C, Bakeera-Kitaka S, Buyze J, Loos J, Buvé A. Factors influencing social self-disclosure among adolescents living with HIV in Eastern Africa. *AIDS Care* [Internet]. 2015 Dec 2 [cited 2019 Nov 12];27(S1):36–46. Available from: 10.1080/09540121.2015.1051501. 10.1080/09540121.2015.1051501 PMC4685614 26616124

[43] Abubakar A, Van de Vijver R, Fischer FJ, Hassan R, Gona AS, Tumaini Dzombo JK. J, et al. ‘Everyone has a secret they keep close to their hearts’: Challenges faced by adolescents living with HIV infection on the Kenyan coast. *BMC Public Health* [Internet]. 2016 Feb 29 [cited 2019 Nov 12];16(1):1–8. Available from: DOI 10.1186/s12889-016-2854-y. 10.1186/s12889-016-2854-y PMC4772469 26927422

[44] Siu GE, Kennedy CE, Bakeera-Kitaka S. Young people with HIV attending a transition clinic in Kampala, Uganda: An exploratory study of social context, illness trajectories, and pathways to HIV testing and treatment. *Child Youth Serv Rev* [Internet]. 2016 Jun 1 [cited 2019 Nov 12];65:9–16. Available from: 10.1016/j.childyouth.2016.03.015.

[45] Folayan MO, Cáceres CF, Sam-Agudu NA, Odetoyinbo M, Stockman JK, Harrison A, et al. Psychological stressors and coping strategies used by adolescents living with and not living with HIV infection in Nigeria. *AIDS Behav* [Internet]. 2017 [cited 2019 Nov 12];21(9):2736–45. Available from: doi: 10.1007/s10461-016-1534-3. 10.1007/s10461-016-1534-3 PMC6510022 27605363

[46] MacCarthy S, Saya U, Samba C, Birungi J, Okoboi S, Linnemayr S. “How am I going to live?”: Exploring barriers to ART adherence among adolescents and young adults living with HIV in Uganda. *BMC Public Health* [Internet]. 2018 [cited 2019 Nov 12];18(1):1158. Available from: 10.1186/s12889-018-6048-7. 10.1186/s12889-018-6048-7 PMC6172755 30286746

[47] Bakeera-Kitaka S, Smekens T, Jespers V, Wobudeya E, Loos J, Colebunders R, et al. Factors influencing the risk of becoming sexually active among HIV infected adolescents in Kampala and Kisumu, East Africa. *AIDS Behav* [Internet]. 2019 [cited 2019 Dec 8];23:1375–86. Available from: 10.1007/s10461-018-2323-y. 10.1007/s10461-018-2323-y 30406334

[48] Kagee A, Coetzee B, Toit S, Du, Loades ME, Coetzee · B, Toit · S, Du, et al. Psychosocial predictors of quality of life among South African adolescents receiving antiretroviral therapy. *Qual Life Res* [Internet]. 2019 Jan [cited 2019 Nov 12];28(1):57–65. Available from: 10.1007/s11136-018-2010-5. 10.1007/s11136-018-2010-5 PMC6754750 30244360

[49] Luseno WK, Iritani BJ, Maman S, Mbai I, Ongili B, Otieno FA, et al. “If the mother does not know, there is no way she can tell the adolescent to go for drugs”: Challenges in promoting health and preventing transmission among pregnant and parenting Kenyan adolescents living with HIV. *Child Youth Serv Rev* [Internet]. 2019 [cited 2019 Nov 12]; 103:100–6. Available from: 10.1016/j.childyouth.2019.05.036. 10.1016/j.childyouth.2019.05.036 PMC6628199 31308586

[50] Toska E, Cluver L, Orkin M, Bains A, Sherr L, Berezin M, et al. Screening and supporting through schools: Educational experiences and needs of adolescents living with HIV in a South African cohort. *BMC Public Health* [Internet]. 2019 [cited 2019 Nov 12];19(272):1–10. Available from: 10.1186/s12889-019-6580-0. 10.1186/s12889-019-6580-0 PMC6404343 30841878

[51] Willis N, Mavhu W, Wogrin C, Mutsinze A, Kagee A. Understanding the experience and manifestation of depression in adolescents living with HIV in Harare, Zimbabwe. *PLoS One* [Internet]. 2018 Jan 3 [cited 2019 Nov 12];13(1):e0190423–e0190423. Available from: 10.1371/journal.pone.0190423.g001. 10.1371/journal.pone.0190423 PMC5752002 29298326

[52] Carbone NB, Njala J, Jackson DJ, Eliya MT, Chilangwa C, Tseka J, et al. “I would love if there was a young woman to encourage us, to ease our anxiety which we would have if we were alone”: Adapting the Mothers2Mothers Mentor Mother Model for adolescent mothers living with HIV in Malawi. *PLoS One* [Internet]. 2019 [cited 2019 Nov 12];14(6):e0217693–e0217693. Available from: 10.1371/journal.pone.0217693. 10.1371/journal.pone.0217693 PMC6555548 31173601

[53] Petersen I, Bhana A, Myeza N, Alicea S, John S, Holst H, et al. Psychosocial challenges and protective influences for socio-emotional coping of HIV + adolescents in South Africa: A qualitative investigation. *AIDS Care - Psychological and Socio-Medical Aspects of AIDS/HIV* [Internet]. 2010 [cited 2020 Mar 3]. p. 970–8. Available from: 10.1080/09540121003623693. 10.1080/09540121003623693 PMC3037257 20229370

[54] Thupayagale-Tshweneagae G, Benedict S. The burden of secrecy among South African adolescents orphaned by HIV and AIDS. *Issues Ment Health Nurs* [Internet]. 2011 [cited 2019 Nov 10];32(6):355–8. Available from: https://www.tandfonline.com/action/journalInformation?journalCode=imhn20. 10.3109/01612840.2011.576128 21692573

[55] Mavhu W, Berwick J, Chirawu P, Copas A, Dirawo J, Willis N (2013). Enhancing psychosocial support for HIV positive adolescents in Harare, Zimbabwe. PLoS ONE.

[56] Kim MH, Mazenga AC, Yu X, Devandra A, Nguyen C, Ahmed S, et al. Factors associated with depression among adolescents living with HIV in Malawi. *BMC Psychiatry* [Internet]. 2015 [cited 2019 Dec 8];15(264):1–12. Available from: doi: 10.1186/s12888-015-0649-9. 10.1186/s12888-015-0649-9 PMC4624356 26503291

[57] Mutumba M, Bauermeister JA, Harper GW, Musiime V, Lepkowski J, Resnicow K, et al. Psychological distress among Ugandan adolescents living with HIV: Examining stressors and the buffering role of general and religious coping strategies. *Glob Public Health* [Internet]. 2017;12(12):1479–91. Available from: doi: 10.1080/17441692.2016.1170871. 10.1080/17441692.2016.1170871 28278753

[58] Kim S, Kim SH, McDonald S, Fidler S, Foster C. Transition to adult services—a positive step. *AIDS Care* [Internet]. 2017;29(7):885–9. Available from: http://search.ebscohost.com.libezproxy.dundee.ac.uk/login.aspx?direct=true&db=jlh&AN=123366777&site=ehost-live&scope=site. 10.1080/09540121.2016.1268672 27973920

[59] Ashaba S, Cooper-Vince C, Maling S, Rukundo GZ, Akena D, Tsai AC. Internalised HIV stigma, bullying, major depressive disorder, and high-risk suicidality among HIV-positive adolescents in rural Uganda. *Heal Sci Kampala, Uganda Glob Ment Heal* [Internet]. 2018 [cited 2019 Dec 8];5:1–10. Available from: doi:10.1017/gmh.2018.15. 10.1017/gmh.2018.15 PMC6036650 29997894

[60] Okawa S, Kabaghe SM, Mwiya M, Kikuchi K, Jimba M, Kankasa C, et al. Psychological well-being and adherence to antiretroviral therapy among adolescents living with HIV in Zambia. *AIDS Care* [Internet]. 2018 May [cited 2019 Nov 12];30(5):634–42. Available from: 10.1080/09540121.2018.1425364. 10.1080/09540121.2018.1425364 29347827

[61] Doku PN. Parental HIV/AIDS status and death, and children’s psychological well-being. *Int J Ment Health Syst* [Internet]. 2009 [cited 2019 Nov 10];3(26):1–8. Available from: doi:10.1186/1752-4458-3-26. 10.1186/1752-4458-3-26 PMC2794254 19930684

[62] Doku PN. Psychosocial adjustment of children affected by HIV/AIDS in Ghana. *J Child Adolesc Ment Health* [Internet]. 2010 [cited 2019 Nov 10];22(1):25–34. Available from: doi.10.2989/17280583.2010.493662. 10.2989/17280583.2010.493662 25859697

[63] Beyeza-Kashesya J, Kaharuza F, Ekström AM, Neema S, Kulane A, Mirembe F. To use or not to use a condom: A prospective cohort study comparing contraceptive practices among HIV-infected and HIV-negative youth in Uganda. *BMC Infect Dis* [Internet]. 2011;11(144):1–11. Available from: http://www.biomedcentral.com/1471-2334/11/144. 10.1186/1471-2334-11-144 PMC3128049 21605418

[64] Demmer C, Rothschild N. Bereavement among South African adolescents following a sibling’s death from AIDS. *African J AIDS Res* [Internet]. 2011 Apr [cited 2019 Oct 21];10(1):15–24. Available from: doi 10.2989/16085906.2011.575544. 10.2989/16085906.2011.575544 PMC3156418 21852964

[65] Mutumba M, Resnicow K, Bauermeister JA, Harper GW, Musiime V, Snow RC, et al. Development of a psychosocial distress measure for Ugandan adolescents living with HIV. *AIDS Behav* [Internet]. 2015;19(2):380–92. Available from: doi.10.1007/s10461-014-0973-y. 10.1007/s10461-014-0973-y 25577026

[66] Wong K, Zucker J, Fernandes H, Cennimo D. Adolescent HIV viral load in an urban hospital in Newark, New Jersey. *Int J Pediatr Adolesc Med* [Internet]. 2016 Sep;3(3):103–8. Available from: http://search.ebscohost.com.libezproxy.dundee.ac.uk/login.aspx?direct=true&db=cmedm&AN=30805478&site=ehost-live&scope=site. 10.1016/j.ijpam.2016.04.001 PMC6372445 30805478

[67] Mutumba M, Bauermeister JA, Harper GW, Musiime V, Lepkowski J, Resnicow K, et al. Psychological distress among Ugandan adolescents living with HIV: Examining stressors and the buffering role of general and religious coping strategies. Glob Public Health. 2016;1–14. 10.1080/17441692.2016.1170871 28278753

[68] Skovdal M, Ogutu VO, Aoro C, Campbell C. Young carers as social actors: Coping strategies of children caring for ailing or ageing guardians in Western Kenya. *Soc Sci Med* [Internet]. 2009;69(4):587–95. Available from: https://www2.scopus.com/inward/record.uri?eid=2-s2.0-67849129019&doi=10.1016%2Fj.socscimed.2009.06.016&partnerID=40&md5=06c2d333cebdc53e4ce74354fe95cf8a. 10.1016/j.socscimed.2009.06.016 19570600

[69] Kim MH, Mazenga AC, Yu X, Ahmed S, Paul ME, Kazembe PN, et al. High self-reported non-adherence to antiretroviral therapy amongst adolescents living with HIV in Malawi: Barriers and associated factors. *J Int AIDS Soc* [Internet]. 2017 [cited 2019 Dec 8];20(21437):1–12. Available from: 10.7448/IAS.20.1.21437. 10.7448/IAS.20.1.21437 PMC5515061 28406275

[70] Kim S-H, McDonald S, Kim S, Foster C, Fidler S. Importance of self-motivation and social support in medication adherence in HIV-infected adolescents in the United Kingdom and Ireland: A multicenter HYPNet study. *AIDS Patient Care STDs* [Internet]. 2015 Jun;29(6):354–64. Available from: http://search.ebscohost.com.libezproxy.dundee.ac.uk/login.aspx?direct=true&db=jlh&AN=103804282&site=ehost-live&scope=site. 10.1089/apc.2014.0335 25825814

[71] Adegoke CO, Steyn MG. A photo voice perspective on factors contributing to the resilience of HIV-positive Yoruba adolescent girls in Nigeria. *J Adolesc* [Internet]. 2017 Apr 1 [cited 2019 Nov 11];56:1–10. Available from: http://search.ebscohost.com.libezproxy.dundee.ac.uk/login.aspx?direct=true&db=jlh&AN=121673179&site=ehost-live&scope=site. 10.1016/j.adolescence.2017.01.003 28110113

[72] Kemigisha E, Zanoni B, Bruce K, Menjivar R, Kadengye D, Atwine D, et al. Prevalence of depressive symptoms and associated factors among adolescents living with HIV/AIDS in Southwestern Uganda. *AIDS Care* [Internet]. 2019 [cited 2019 Nov 11];31(10):1297–303. Available from: 10.1080/09540121.2019.1566511. 10.1080/09540121.2019.1566511 PMC6614012 30621430

[73] Hodgson I, Ross J, Haamujompa C, Gitau-Mburu D. Living as an adolescent with HIV in Zambia–lived experiences, sexual health, and reproductive needs. *AIDS Care* [Internet]. 2012 [cited 2019 Jun 7];24(10):1204–10. Available from: 10.1080/09540121.2012.658755. 10.1080/09540121.2012.658755 22380932

[74] Mutumba M, Musiime V, Lepkwoski JM, Harper GW, Snow RC, Resnicow K, et al. Examining the relationship between psychological distress and adherence to anti-retroviral therapy among Ugandan adolescents living with HIV. *AIDS Care - Psychol Socio-Medical Asp AIDS/HIV* [Internet]. 2016 [cited 2019 Nov 12];28(7):807–15. Available from: 10.1080/09540121.2015.1131966. 10.1080/09540121.2015.1131966 27294696

[75] Ashaba S, Kaida A, Burns BF, O’Neil K, Dunkley E, Psaros C, et al. Understanding coping strategies during pregnancy and the postpartum period: A qualitative study of women living with HIV in rural Uganda. *BMC Pregnancy Childbirth* [Internet]. 2017;17(1):138. Available from: doi: 10.1186/s12884-017-1321-9. 10.1186/s12884-017-1321-9 PMC5423027 28482821

[76] Loades ME, Kagee A. Exploring our understanding of fatigue among adolescents living with HIV: Highlighting the unknown. *J Health Psychol* [Internet]. 2019 Jan;24(1):125–36. Available from: doi:10.1177/1359105317710320. 10.1177/1359105317710320 PMC6084770 28810460

[77] Toska E, Cluver LD, Hodes R, Kidia KK. Sex and secrecy: How HIV-status disclosure affects safe sex among HIV-positive adolescents. *AIDS Care - Psychol Socio-Medical Asp AIDS/HIV* [Internet]. 2015 [cited 2019 Jun 25];27(S1):47–58. Available from: 10.1080/09540121.2015.1071775. 10.1080/09540121.2015.1071775 PMC4699474 26616125

[78] Hapazari J. The nexus of decision making, re-allocation and migration on children orphaned and affected by HIV and AIDS: a case study of rural Lesotho. *African Sociol Rev* [Internet]. 2020;24(2):212–32. Available from: https://www.jstor.org/stable/10.2307/48630999.

[79] Mutumba M, Bauermeister JA, Harper GW, Musiime V, Lepkowski J, Resnicow K, et al. Psychological distress among Ugandan adolescents living with HIV: Examining stressors and the buffering role of general and religious coping strategies. *Glob Public Health* [Internet]. 2016;12(12):1479–91. Available from: 10.1080/17441692.2016.1170871. 10.1080/17441692.2016.1170871 28278753

[80] Wong VJ, Murray KR, Phelps R, Vermund SH, Mccarraher DR. Adolescents, young people, and the 90-90-90 goals: A call to improve HIV testing and linkage to treatment. *AIDS* [Internet]. 2017 [cited 2019 Jun 6];31(Suppl3):S191–4. Available from: doi: 10.1097/QAD.0000000000001539. 10.1097/QAD.0000000000001539 PMC5497776 28665876

[81] Carey KB, Guthrie KM, Rich CM, Krieger NH, Norris AL, Kaplan C, et al. Alcohol use and sexual risk behaviour in young women: A qualitative study. *AIDS Behav* [Internet]. 2019 [cited 2021 Aug 21];23(6):1647–55. Available from: doi:10.1007/s10461-018-2310-3. 10.1007/s10461-018-2310-3 PMC6461532 30311105

[82] Senn TE, Carey MP, Vanable PA (2010). The intersection of violence, substance use, depression, and STDs: Testing of a syndemic pattern among patients attending an urban STD clinic. J Natl Med Assoc.

[83] Kim JY, Yang Y, Kim HK. The impact of alcohol use on antiretroviral therapy adherence in Koreans living with HIV. *Asian Nurs Res* (Korean Soc Nurs Sci) [Internet]. 2018 [cited 2021 Aug 21];12(4):258–64. Available from: 10.1016/J.ANR.2018.10.002. 10.1016/j.anr.2018.10.002 30316838

[84] Nash K, O’Malley G, Geoffroy E, Schell E, Bvumbwe A, Denno DM. “Our girls need to see a path to the future”: Perspectives on sexual and reproductive health information among adolescent girls, guardians, and initiation counsellors in Mulanje district, Malawi. *Reprod Health* [Internet]. 2019 [cited 2021 Aug 23];16(8):1–13. Available from: 10.1186/s12978-018-0661-x. 10.1186/s12978-018-0661-x PMC6347744 30683127

[85] Adegoke CO, Steyn MG. Yoruba culture and the resilience of HIV-positive adolescent girls in Nigeria. *Cult Health Sex* [Internet]. 2018 Nov 2 [cited 2019 Jun 28];20(11):1287–98. Available from: https://www.tandfonline.com/doi/full/10.1080/13691058.2017.1422806. 10.1080/13691058.2017.1422806 29388478

[86] Kalichman SC, Washington C, Grebler T, Hoyt G, Welles B, Kegler C (2015). Medication adherence and health outcomes of people living with HIV who are food insecure and prescribed antiretrovirals that should be taken with food. Infect Dis Ther.

[87] Singer A, Weiser S, McCoy S. Does food insecurity undermine adherence to antiretroviral therapy? A systematic review. AIDS Behav [Internet]. 2015;19(8):1510–26. Available from: doi:10.1007/s10461-014-0873-1. 10.1007/s10461-014-0873-1 25096896

[88] Oluma A, Abadiga M, Mosisa G, Etafa W, Fekadu G (2020). Food insecurity among people living with HIV/AIDS on ART Follower at Public Hospitals of Western Ethiopia. Int J Food Sci.

[89] Chop E, Duggaraju A, Malley A, Burke V, Caldas S, Yeh PT (2017). Food insecurity, sexual risk behavior and adherence to antiretroviral therapy among women living with HIV: A systematic review. Health Care Women Int.

[90] Boyle MP. Enacted stigma and felt stigma experienced by adults who stutter. *J Commun Disord* [Internet]. 2018;73(August 2017):50–61. Available from: 10.1016/j.jcomdis.2018.03.004. 10.1016/j.jcomdis.2018.03.004 29574262

[91] Arigo D, Suls, Joshua JM, Smyth MM. Social comparisons and chronic illness: Research synthesis and clinical implications. *Health Psychol Rev* [Internet]. 2012 [cited 2021 Jun 2];8(2):154–214. Available from: https://www.tandfonline.com/action/journalInformation?journalCode=rhpr20. 10.1080/17437199.2011.634572 25053133

[92] Coetzee BJ, Loades ME, Stefani ·, Toit D, Kagee · Ashraf Du, Toit S, et al. Correlates of fatigue among South African adolescents living with HIV and receiving antiretroviral therapy. *AIDS Behav* [Internet]. 2019 Mar [cited 2019 Nov 12];23(3):1–15. Available from: 10.1007/s10461-018-02384-6. 10.1007/s10461-018-02384-6 PMC6754751 30659425

[93] Kim WJ, Ku NS, Lee Y-J, Ahn JY, Kim SB, Ahn H-W, et al. Utility of the Montreal Cognitive Assessment (MoCA) and its subset in HIV-associated neurocognitive disorder (HAND) screening. *J Psychosom Res* [Internet]. 2016 Jan;80:53–7. Available from: http://search.ebscohost.com.libezproxy.dundee.ac.uk/login.aspx?direct=true&db=jlh&AN=111929466&site=ehost-live&scope=site. 10.1016/j.jpsychores.2015.11.006 26721548

[94] Proudfoot JG, Jayawant A, Whitton AE, Parker G, Manicavasagar V, Smith M (2012). Mechanisms underpinning effective peer support: A qualitative analysis of interactions between expert peers and patients newly diagnosed with bipolar disorder. BMC Psychiatry.

[95] Mackworth-young CRS, Bond V, Wringe A, Bond V, Wringe A, Bond V. Secrets and silence: Agency of young women managing HIV disclosure. *Med Anthropol* [Internet]. 2020;39(8):720–34. Available from: 10.1080/01459740.2020.1764551. 10.1080/01459740.2020.1764551 32469242

[96] Callaghan JEM, Gambo Y, Fellin LC (2015). Hearing the silences: Adult Nigerian women’s accounts of ‘early marriages’. Fem Psychol.

[97] Dale S, Cohen M, Weber K, Cruise R, Kelso G, Brody L. Abuse and resilience in relation to HAART medication adherence and HIV viral load among women with HIV in the United States. *AIDS Patient Care STDs* [Internet]. 2014 Mar;28(3):136–43. Available from: doi:10.1089/apc.2013.0329. 10.1089/apc.2013.0329 PMC3948478 24568654

[98] Kralik D, Van Loon A, Visentin K. Resilience in the chronic illness experience. *Educ Action Res* [Internet]. 2006 [cited 2021 Aug 21];14(2):187–201. Available from: 10.1080/09650790600718035.

[99] Bandura A. Self-efficacy: Toward a unifying theory of behavioral change. *Pyschological Rev* [Internet]. 1977 [cited 2021 Aug 21];84(2):191–215. Available from: https://psycnet.apa.org/fulltext/1977-25733-001.pdf. 10.1037//0033-295x.84.2.191 847061

[100] Tuthill EL, Miller JD, Collins SM, Widen EM, Onono M, Young SL. HIV infection, hunger, breastfeeding self-efficacy, and depressive symptoms are associated with exclusive breastfeeding to six months among women in western Kenya: A longitudinal observational study. *Int Breastfeed J* [Internet]. 2020 [cited 2021 Aug 22];15(4):1–9. Available from: 10.1186/s13006-019-0251-8. 10.1186/s13006-019-0251-8 PMC6966845 31948438

[101] Doherty T, Sanders D, Goga A, Jackson D (2011). Implications of the new WHO guidelines on HIV and infant feeding for child survival in South Africa. Bull World Health Organ.

[102] Nyogea D, Mtenga S, Henning L, Franzeck FC, Glass TR, Letang E (2015). Determinants of antiretroviral adherence among HIV positive children and teenagers in rural Tanzania: A mixed methods study. BMC Infect Dis.

[103] Doku PN, Dotse JE, Mensah KA. Perceived social support disparities among children affected by HIV/AIDS in Ghana: A cross-sectional survey. *BMC Public Health* [Internet]. 2015;15(1):1–10. Available from: 10.1186/s12889-015-1856-5. 10.1186/s12889-015-1856-5 PMC4457987 26048140

[104] Mbonu NC, Van Den Borne B, De Vries NK, Gonzalez J-P. Stigma of People with HIV/AIDS in Sub-Saharan Africa: A literature review. J Trop Med. 2009;1–14. 10.1155/2009/145891 PMC2836916 20309417

[105] Singh V, Lata S (2018). A systematic review of HIV/AIDS-related stigma among children and youth living with HIV. IAHRW Int J Soc Sci Rev.

[106] *Vulnerable Child Youth Stud*. 2018;13(3):228–38. 10.1080/17450128.2018.1439211 PMC6075833 30083221

[107] *AIDS Behav* [Internet]. 2021;25(2):344–53. Available from: https://doi.org/10.1007/s10461-020-02974-3.

[108] Chi P, Li X. Impact of parental HIV/AIDS on children’s psychological well-being: A systematic review of global literature. *AIDS Behav* [Internet]. 2013 Sep [cited 2020 Feb 11]; 17(7):2554–74. Available from: DOI 10.1007/s10461-012-0290-2. 10.1007/s10461-012-0290-2 PMC3568204 22972606

[109] Kumar P, Kumar A, Sp R, Srinivas V, Dandona R (2016). A comparative assessment of generalized anxiety, conduct, and peer relationship problems among AIDS and other orphaned children in India. BMC Psychiatry.

[110] McCann BR, Roberto KA (2012). Relational systems: How older women with chronic health problems construct close relationships. J Fam Issues.

[111] Remien RH, Stirratt MJ, Nguyen N, Robbins RN, Pala AN, Mellins CA (2019). Mental health and HIV/AIDS: The need for an integrated response. AIDS.

[112] Bukenya D, Mayanja BN, Nakamanya S, Muhumuza R, Seeley J. What causes non-adherence among some individuals on long-term antiretroviral therapy? Experiences of individuals with poor viral suppression in Uganda. *AIDS Res Ther* [Internet]. 2019;16(1):1–9. Available from: 10.1186/s12981-018-0214-y. 10.1186/s12981-018-0214-y PMC6340167 30665440

[113] Mthiyane N, Harling G, Chimbindi N, Baisley K, Seeley J, Dreyer J (2021). Common mental disorders and HIV status in the context of DREAMS among adolescent girls and young women in rural KwaZulu-Natal, South Africa. BMC Public Health.

[114] Melesse DY, Mutua MK, Choudhury A, Wado YD, Faye CM, Neal S (2020). Adolescent sexual and reproductive health in sub-Saharan Africa: Who is left behind?. BMJ Glob Heal.

[115] Han H, Yang F, Murray S, Mbita G, Bangser M, Rucinski K, et al. Characterizing a sexual health and HIV risk stratification scale for sexually active adolescent girls and young women (AGYW) in Tanzania. *PLoS One* [Internet]. 2021;16(3):1–15. Available from: 10.1371/journal.pone.0248153. 10.1371/journal.pone.0248153 PMC7971553 33735253

[116] Hopkins J, Collins L (2017). How linked are national HIV and SRHR strategies? A review of SRHR and HIV strategies in 60 countries. Health Policy Plan.

[117] WHO. SRH and HIV Linkages Compendium: Indicators and Related Assessment Tools [Internet]. IPPF, UNFPA, and. London WHO. 2014. Available from: http://www.unfpa.org/publications/srh-and-hiv-linkages-compendium-indicators-and-related-assessment-tools.

[118] Roxo U, Mobula ML, Walker D, Ficht A, Yeiser S, Linda Mobula M, et al. Prioritizing the sexual and reproductive health and rights of adolescent girls and young women within HIV treatment and care services in emergency settings: A girl-centered agenda. *Reprod Health* [Internet]. 2019 [cited 2019 Nov 11];16((Suppl 1)15):1–13. Available from: 10.1186/s12978-019-0710-0. 10.1186/s12978-019-0710-0 PMC6538549 31138222

[119] Morris JL, Rushwan H. Adolescent sexual and reproductive health: The global challenges. *Int J Gynecol Obstet* [Internet]. 2015 [cited 2020 Mar 28];131:S40–2. Available from: doi: 10.1016/j.ijgo.2015.02.006. 10.1016/j.ijgo.2015.02.006 26433504

[120] Wamoyi J, Buller AM, Nyato D, Kyegombe N, Meiksin R, Heise L. “Eat and you will be eaten”: A qualitative study exploring costs and benefits of age-disparate sexual relationships in Tanzania and Uganda: implications for girls’ sexual and reproductive health interventions. *Reprod Health* [Internet]. 2018 Dec 13;15(1):1–11. Available from: doi: 10.1186/s12978-018-0650-0. 10.1186/s12978-018-0650-0 PMC6293630 30545378

[121] Goffman E (1963). Stigma: Notes on the management of spoiled identity.

[122] Overstreet NM, Quinn DM. The intimate partner violence stigmatization model and barriers to help seeking. *Basic Appl Soc Psych* [Internet]. 2013 [cited 2021 Sep 13];35(1):109–22. Available from: doi.10.1080/01973533.2012.746599. 10.1080/01973533.2012.746599 PMC3601798 23524454

[123] Pryor JB, Reeder GD, Hall JC (2011). HIV-related stigma. HIV/AIDS in the post-HAART era: Manifestations, treatment, and epidemiology.

[124] Tran BX, Than PQT, Tran TT, Nguyen CT, Latkin CA. Changing sources of stigma against patients with HIV/AIDS in the rapid expansion of antiviral treatment services in Vietnam. *Biomed Res Int* [Internet]. 2019;1–9. Available from: doi: 10.1155/2019/4208638. 10.1155/2019/4208638 PMC6363237 30805364

[125] Gostin LO, Hodge JG (1998). Piercing the veil of secrecy in HIV / AIDS and other sexually transmitted diseases: Theories of privacy and disclosure in partner notification – contact tracing, the “right to know,” and the " duty to warn”. Introduction: The three meanings of partner. Duke J Gend Law Policy.

[126] Zhang L, Li X, Kaljee L, Fang X, Lin X, Zhao G (2009). “I felt I have grown up as an adult”: Caregiving experience of children affected by HIV/AIDS in China. Child Care Health Dev.

[127] Bos AER, Pryor JB, Reeder GD, Stutterheim SE (2013). Stigma: Advances in theory and research. Basic Appl Soc Psych.

[128] Dijker AJ, Koomen W. Extending Weiner’s Attribution-Emotion Model of Stigmatization of Ill Persons. *Basic Appl Soc Psych* [Internet]. 2003 [cited 2021 Sep 14];25(1):51–68. Available from: doi.10.1207/S15324834BASP2501_4.

[129] Hedge B, Devan K, Catalan J, Cheshire A, Ridge D. HIV-related stigma in the UK then and now: to what extent are we on track to eliminate stigma? A qualitative investigation. *BMC Public Health* [Internet]. 2021 [cited 2021 Aug 23];21(1022):1–10. Available from: 10.1186/s12889-021-11000-7. 10.1186/s12889-021-11000-7 PMC8166014 34053441

[130] Sixsmith J, Fang ML, Woolrych R, Sixsmith A. Applying a multidimensional intersectionality analysis: Perspectives of sense of place from low-income seniors at the intersections of multiple oppressions, positionalities, and identities [Internet]. *International Conference on Public Policy* (ICPP). 2015. Available from: https://www.ippapublicpolicy.org/file/paper/1435799000.pdf.

[131] Ussher JM, Perz J, Metusela C, Hawkey AJ, Morrow M, Narchal R (2017). Negotiating discourses of shame, secrecy, and silence: migrant and refugee women’s experiences of sexual embodiment. Arch Sex Behav.

[132] Samuels A. Strategies of silence in an age of transparency: Navigating HIV and visibility in Aceh, Indonesia. *Hist Anthropol Chur* [Internet]. 2020;0(0):1–18. Available from: 10.1080/02757206.2020.1830384.

[133] Baxen J, Haipinge E. School experiences of HIV-positive secondary school learners on ARV treatment in Namibia. *Int J Educ Dev* [Internet]. 2015;41:237–44. Available from: 10.1016/j.ijedudev.2014.11.002.

[134] Pantelic M, Sprague L, Stangl AL (2019). It’s not “all in your head”: Critical knowledge gaps on internalized HIV stigma and a call for integrating social and structural conceptualizations. BMC Infect Dis.

[135] Abrams K (1999). From autonomy to agency: Feminist perspectives on self-direction. Fem Perspect Self-Direction.

[136] Goodkind S, Brinkman BG, Elliott K, Goodkind S, Brinkman BG, Redefining KE, et al. Redefining resilience and reframing resistance: Empowerment programming with Black girls to address societal inequities. *Behav Med* [Internet]. 2020;46(3–4):317–29. Available from: 10.1080/08964289.2020.1748864. 10.1080/08964289.2020.1748864 32787728

[137] Beyeza-Kashesya J, Kaharuza F, Ekström AM, Neema S, Kulane A, Mirembe F, et al. To use or not to use a condom: A prospective cohort study comparing contraceptive practices among HIV-infected and HIV-negative youth in Uganda. *BMC Infect Dis* [Internet]. 2011 Jan [cited 2019 Nov 10];11(1):144. Available from: http://www.biomedcentral.com/1471-2334/11/144. 10.1186/1471-2334-11-144 PMC3128049 21605418

[138] Crenshaw K. Demarginalising the intersection of race and sex: A Black feminist critique of antidiscrimination doctrine, feminist theory and antiracist politics. *Univ Chicago Leg Forum* [Internet]. 1989;1989(1):139–68. Available from: http://chicagounbound.uchicago.edu/uclf/vol1989iss1/8.

[139] Carastathis A. The concept of intersectionality in feminist theory. *Philos Compass* [Internet]. 2014 [cited 2021 Aug 4];9:304–14. Available from: doi:10.1111/phc3.12129.

[140] Rifkin SB (2014). Examining the links between community participation and health outcomes: A review of the literature. Health Policy Plan.

[141] Francés F, La Parra Casado D. Participation as a driver of health equity [Internet]. World Health Organization Europe. 2019. Available from: http://rua.ua.es/dspace.

[142] Grocott A, McSherry W. The patient experience: Informing practice through identification of meaningful communication from the patient’s perspective. *Healthcare* [Internet]. 2018 [cited 2021 Mar 23];6(26):1–14. Available from: www.mdpi.com/journal/healthcare. 10.3390/healthcare6010026 PMC5872233 29558392

[143] Rai SS, Peters RMH, Syurina EV, Irwanto I, Naniche D, Zweekhorst MBM. Intersectionality and health-related stigma: Insights from experiences of people living with stigmatized health conditions in Indonesia. *Int J Equity Health* [Internet]. 2020 [cited 2021 Oct 2];19(206):1–15. Available from: 10.1186/s12939-020-01318-w. 10.1186/s12939-020-01318-w PMC7661268 33176809

[144] Amuche NJ, Emmanuel EI, Innocent NE. HIV/AIDS in sub-Saharan Africa: Current status, challenges, and prospects. *Asian Pacific J Trop Dis* [Internet]. 2017;7(4):239–56. Available from: doi:10.12980/apjtd.7.2017D6-366.

[145] Corno L, de Walque D. Mines, migration, and HIV/AIDS in Southern Africa. *J Afr Econ* [Internet]. 2012;21(3):465–98. Available from: doi:10.1093/jae/ejs005.

[146] Harrison SE, Li X, Vermund SH (2019). From surviving to thriving: The role of resilience in meeting global HIV goals. AIDS.

[147] Sutton-Brown CA. Photovoice: A Methodological Guide. *Photogr Cult* [Internet]. 2014;7(2):169–85. Available from: 10.2752/175145214X13999922103165.

[148] Neimann Rasmussen L, Montgomery P (2018). The prevalence of and factors associated with the inclusion of non-English language studies in Campbell systematic reviews: A survey and meta-epidemiological study. Syst Rev.

[149] Reynolds J, Kizito J, Ezumah N, Mangesho P, Allen E, Chandler C. Quality assurance of qualitative research: a review of the discourse. *Heal Res Policy Syst* [Internet]. 2011;9(43):1–10. Available from: http://www.health-policy-systems.com/content/9/1/43. 10.1186/1478-4505-9-43 PMC3267652 22182674

