# Supplementary figures and images for: Adolescent Girls and Young Women’s Experiences of Living with HIV in the Context of Patriarchal Culture in Sub-Saharan Africa: A Scoping Review
Source: AIDS Behav. 2022 Nov 1;27(5):1365–79. doi: 10.1007/s10461-022-03872-6 (PMC10129999; doi:10.1007/s10461-022-03872-6)

**Figure 1: PRISMA flow chart**


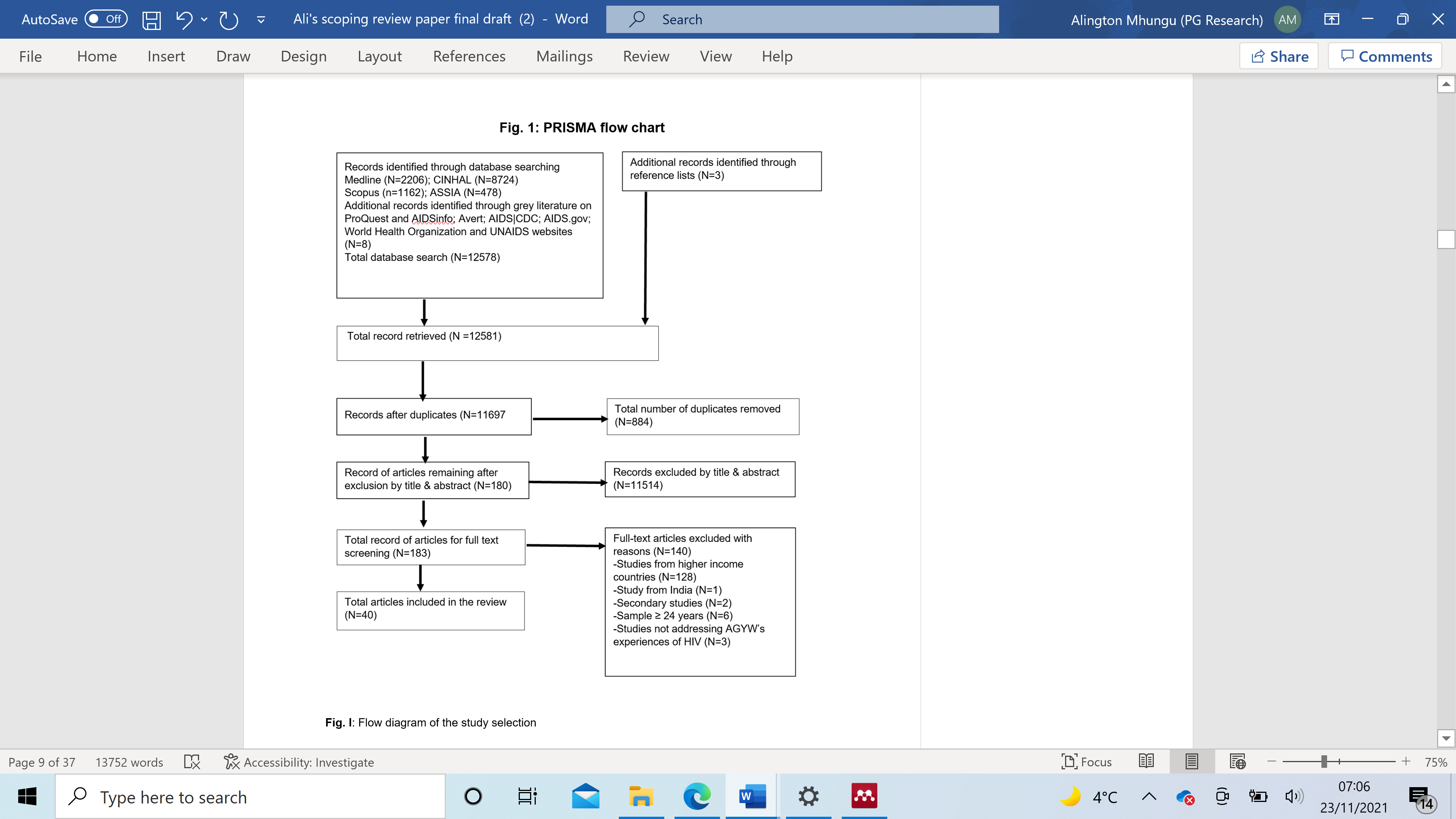

Supplement: Supplementary file 1 — Supplementary Material 1 [file 10461_2022_3872_MOESM1_ESM.docx]
