# Supplementary material for: Adolescent Girls and Young Women’s Experiences of Living with HIV in the Context of Patriarchal Culture in Sub-Saharan Africa: A Scoping Review
Source: AIDS Behav. 2022 Nov 1;27(5):1365–79. doi: 10.1007/s10461-022-03872-6 (PMC10129999; doi:10.1007/s10461-022-03872-6)
Supplement: Supplementary file 2 — Supplementary Material 2 [file 10461_2022_3872_MOESM2_ESM.docx]

| **Population**  “Young femal*”  “Adolescent girls”  “Young women”  “Young adults”  “Teenager” | S1 “young women”; S2 “young femal*”; S3 “adolescents or teenagers or young adults or teen or youth”; S4 “adolescent girls” OR “teenagers” OR “young adults” OR” teen” OR “youth”  S1 AND S3; S1 AND S4; S1 OR S3; S1 OR S4 |
| --- | --- |
| **Concept**  “HIV”  “AIDS”  “HIV/AIDS” | S5 “HIV”; S6 “AIDS”; S7 “HIV/AIDS”; S8 “HIV” OR “AIDS” OR “AIDS” OR “acquired human immunodeficiency syndrome” OR “human immunodeficiency virus”; S9 “living with HIV/AIDS”; S8 AND S9; S8 OR S9 |
| **Context**  “Sub-Saharan Africa”  “Patriarchy”  “Participatory research” | S10 “sub-Saharan Africa” OR “sub-Sahara”; “S13 participation”; “S12 participatory action research or community based participatory research or participatory”  S11 “patriarchal”; “S10 AND S11”; “S10 OR S11”; “S10 AND S11 AND S12”; “S10 OR S11 OR S12”; “S10 AND S11 OR S12” |

**Appendix 1: Search terms used for all databases using Population, Concept, and Context approach**
