# Supplementary material for: Adolescent Girls and Young Women’s Experiences of Living with HIV in the Context of Patriarchal Culture in Sub-Saharan Africa: A Scoping Review
Source: AIDS Behav. 2022 Nov 1;27(5):1365–79. doi: 10.1007/s10461-022-03872-6 (PMC10129999; doi:10.1007/s10461-022-03872-6)
Supplement: Supplementary file 3 — Supplementary Material 3 [file 10461_2022_3872_MOESM3_ESM.docx]

**Appendix II: Quality Assessment Tool (QATSDD) Scores for Reviewed Studies (scores range 0-3)**

| **Criteria/ items** | Item 1 | Item 2 | Item 3 | Item 4 | Item 5 | Item 6 | Item 7 | Item 8 | Item 9 | Item 10 | Item 11 | Item 12 | Item 13 | Item 14 | Item 15 | Item 16 | **Score per study** |
| --- | --- | --- | --- | --- | --- | --- | --- | --- | --- | --- | --- | --- | --- | --- | --- | --- | --- |
| **Studies** |  |  |  |  |  |  |  |  |  |  |  |  |  |  |  |  |  |
| Busza et al., 2013 | 0 | 2 | 3 | 2 | 2 | 3 | 2 | 3 | n/a | n/a | 2 | n/a | 0 | 0 | 0 | 1 | 20/42 (48%) |
| Buyeza. Et al., 2011 | 0 | 2 | 3 | 3 | 1 | 3 | 3 | 3 | 0 | 3 | n/a | 2 | 0 | n/a | 0 | 2 | 25/42 (62%) |
| Adegoke et al., 2017 | 3 | 1 | 2 | 2 | 1 | 1 | 2 | 1 | n/a | n/a | 3 | n/a | 0 | 1 | 1 | 2 | 20/42 (48%) |
| Demmer et al., 201 | 0 | 1 | 0 | 3 | 2 | 2 | 2 | 2 | n/a | n/a | 2 | n/a | 3 | 3 | 0 | 2 | 22/42 (52%) |
| Abubakar et al., 2016 | 0 | 3 | 2 | 3 | 1 | 3 | 1 | 3 | n/a | n/a | 3 | n/a | 0 | 1 | 0 | 2 | 22/42 (52%) |
| MacCarthy et al., 2018 | 0 | 2 | 3 | 2 | 1 | 2 | 2 | 3 | n/a | n/a | 2 | n/a | 2 | 3 | 1 | 3 | 26/42 (62%) |
| Kagee et al., 2019 | 1 | 1 | 1 | 0 | 1 | 3 | 3 | 3 | 3 | 2 | n/a | 3 | 2 | n/a | 0 | 2 | 24/42 (52%) |
| Luseno et al., 2019 | 0 | 3 | 3 | 1 | 2 | 2 | 0 | 3 | n/a | n/a | 2 | n/a | 0 | 2 | 1 | 3 | 21/42 (52%) |
| Lowenthal et al., 2014 | 0 | 1 | 1 | 0 | 1 | 3 | 3 | 3 | n/a | n/a | 0 | n/a | 0 | 2 | 1 | 0 | 18/42 (38%) |
| Kiddia et al., 2014 | 0 | 3 | 2 | 3 | 1 | 2 | 2 | 3 | n/a | n/a | 2 | n/a | 2 | 2 | 1 | 2 | 27/42 (62%) |
| Kemigisha et al., 2019 | 3 | 3 | 2 | 3 | 1 | 3 | 2 | 3 | 2 | 3 | n/a | 3 | 2 | n/a | 0 | 2 | 32/42 (76%) |
| Doku et al., 2010 | 0 | 1 | 1 | 2 | 1 | 3 | 3 | 2 | 1 | 3 | n/a | 3 | 0 | n/a | 2 | 2 | 23/42 (55%) |
| Doku et al., 2009 | 0 | 2 | 3 | 2 | 1 | 2 | 3 | 3 | 1 | 2 | n/a | 2 | 0 | n/a | 0 | 3 | 24/42 (57%) |
| Carbone et al., 2019 | 0 | 1 | 3 | 2 | 2 | 3 | 1 | 2 | n/a | n/a | 2 | n/a | 1 | 2 | 1 | 2 | 22/42 (52%) |
| Hodgson et al., 2012 | 0 | 3 | 2 | 3 | 1 | 2 | 2 | 3 | n/a | n/a | 3 | n/a | 2 | 2 | 0 | 2 | 25/42 (60%) |
| Enimil et al., 2015 | 0 | 2 | 2 | 0 | 2 | 3 | 1 | 2 | 1 | 2 | 2 | 2 | 0 | 0 | 0 | 0 | 18/48 (38%) |
| Madiba et al., 2017 | 0 | 2 | 3 | 3 | 3 | 2 | 2 | 2 | 2 | 2 | n/a | 3 | 0 | n/a | 0 | 2 | 26/42 (62%) |
| Mutumba et al., 2016 | 2 | 2 | 2 | 3 | 2 | 3 | 3 | 3 | n/a | n/a | 3 | n/a | 3 | 2 | 0 | 3 | 31/42 (74%) |
| Willis et al., 2017 | 0 | 2 | 2 | 1 | 1 | 3 | 2 | 3 | n/a | n/a | 2 | n/a | 2 | 3 | 0 | 3 | 24/42 (57%) |
| Zamudio-Haas et al., 2012 | 0 | 2 | 2 | 1 | 2 | 3 | 3 | 3 | n/a | n/a | 3 | n/a | 3 | 0 | 0 | 3 | 25/42 (60%) |
| Mutwa et al., 2013 | 0 | 2 | 2 | 1 | 1 | 3 | 2 | 3 | n/a | n/a | 1 | n/a | 2 | 0 | 0 | 2 | 19/42 (45%) |
| Mutumba et al 2016 b | 3 | 2 | 2 | 1 | 1 | 3 | 0 | 2 | n/a | n/a | 3 | n/a | 1 | 3 | 0 | 3 | 24/42 (57%) |
| Nkwata et al., 2017 | 0 | 2 | 2 | 1 | 1 | 3 | 2 | 3 | 3 | 3 | n/a | 3 | 2 | n/a | 2 | 2 | 29/42 (69%) |
| Mutumba et al., 2015 | 1 | 2 | 2 | 3 | 1 | 3 | 1 | 3 | n/a | n/a | 2 | n/a | 3 | 3 | 0 | 0 | 24/42 (57%) |
| Bekera-Kitaka et al., 2019 | 0 | 3 | 2 | 1 | 1 | 2 | 2 | 0 | 2 | 3 | n/a | 3 | 2 | n/a | 2 | 0 | 23/42 (55%) |
| Nostlinger et al., 2015 | 0 | 3 | 3 | 1 | 3 | 2 | 1 | 3 | 1 | 2 | n/a | 2 | 3 | n/a | 0 | 3 | 27/42 (62%) |
| Mburu et al., 2014 | 0 | 2 | 3 | 2 | 2 | 3 | 2 | 3 | n/a | n/a | 2 | n/a | 1 | 3 | 0 | 0 | 23/42 (55%) |
| Toska et al., 2019 | 0 | 3 | 3 | 2 | 2 | 3 | 1 | 3 | 3 | 3 | n/a | 3 | 1 | n/a | 0 | 3 | 30/42 (71%) |
| Skovdal & Ogutu 2009 | 3 | 3 | 3 | 2 | 1 | 2 | 3 | 3 | n/a | n/a | 3 | n/a | 2 | 1 | 0 | 0 | 22/42 ()52% |
| Sui et al., 2016 | 0 | 2 | 3 | 1 | 1 | 3 | 1 | 3 | n/a | n/a | 3 | n/a | 2 | 3 | 0 | 0 | 22/42 (52%) |
| Wong et al., 2016 | 0 | 3 | 3 | 1 | 2 | 3 | 1 | 3 | 0 | 2 | n/a | 3 | 1 | n/a | 0 | 2 | 24/42 (57%) |
| Kim et al., 2017 | 0 | 3 | 2 | 1 | 2 | 2 | 2 | 1 | 2 | 3 | n/a | 3 | 3 | n/a | 0 | 3 | 27/42 (62%) |
| Thupayangale. et al., 2011 | 1 | 2 | 3 | 1 | 1 | 3 | 2 | 2 | n/a | n/a | 1 | n/a | 1 | 1 | 0 | 2 | 20/42 (48%) |
| Madiba &Josiah 2019 | 0 | 3 | 3 | 1 | 2 | 2 | 2 | 3 | n/a | n/a | 3 | n/a | 2 | 2 | 0 | 3 | 26/42 (62%) |
| Okawa et al., 2018 | 0 | 2 | 3 | 2 | 2 | 2 | 2 | 3 | 0 | 3 | 2 | 2 | 3 | 0 | 3 | 2 | 31/48 (65%) |
| Mavhu et al., 2013 | 0 | 2 | 3 | 1 | 2 | 3 | 3 | 2 | 1 | 2 | 2 | 2 | 0 | 0 | 2 | 0 | 24/48 (50%) |
| Mutumba et al., 2017 | 1 | 3 | 3 | 3 | 1 | 3 | 2 | 3 | n/a | n/a | 3 | n/a | 2 | 0 | 2 | 3 | 29/42 (69%) |
| Petersen et al., 2010 | 0 | 3 | 3 | 1 | 2 | 3 | 1 | 3 | n/a | n/a | 3 | n/a | 2 | 3 | 0 | 3 | 27/42 (64%) |
| Ashaba et al., 2018 | 0 | 2 | 3 | 3 | 2 | 2 | 1 | 2 | 0 | 2 | n/a | 3 | 2 | n/a | 0 | 2 | 24/42 (57%) |
| Kim et al., 2015 | 0 | 3 | 2 | 1 | 2 | 2 | 1 | 2 | 2 | 3 | n/a | 3 | 3 | n/a | 0 | 2 | 26/42 (62%) |
| **Total score per item** | 18/120 | 89/120 | 95/120 | 69/120 | 61/120 | 103/120 | 73/120 | 103/120 | 24/54 | 46/54 | 59/72 | 47/54 | 60/120 | 40/72 | 19/120 | 76/120 |  |
| **Total score per item %** | 15% | 74% | 79% | 58% | 51% | 86% | 61% | 86% | 44% | 85% | 82% | 87% | 50% | 56% | 16% | 63% |  |

**Key**

Item 1: Explicit theoretical framework

Item 2: Statement of aims/objectives in main report

Item 3: Clear description of research setting

Item 4: Evidence of sample size considered in terms of analysis

Item 5: Representative sample of target group of a reasonable size

Item 6: Description of procedure for data collection

Item 7: Rationale for choice of data collection tool(s)

Item 8: Detailed recruitment data

Item 9: Statistical assessment of reliability and validity of measurement tool(s) (Quantitative studies only)

Item 10: Fit between research question and method of data collection (Quantitative studies only)

Item 11: Fit between research question and format and content of data collection tool e.g. interview schedule (Qualitative studies only)

Item 12: Fit between research question and method of analysis (Quantitative studies only)

Item 13: Good justification for analytic method selected

Item 14: Assessment of reliability of analytic process (Qualitative studies only)

Item 15: Evidence of user involvement in design

Item 16: Strengths and limitations critically discussed
