# Supplementary material for: Adolescent Girls and Young Women’s Experiences of Living with HIV in the Context of Patriarchal Culture in Sub-Saharan Africa: A Scoping Review
Source: AIDS Behav. 2022 Nov 1;27(5):1365–79. doi: 10.1007/s10461-022-03872-6 (PMC10129999; doi:10.1007/s10461-022-03872-6)
Supplement: Supplementary file 4 — Supplementary Material 4 [file 10461_2022_3872_MOESM4_ESM.docx]

**Appendix 3: Codes, sub-themes, and overarching themes from AGYW’s experiences of HIV/AIDS**

**What do we know about the experiences of living with or being affected by HIV/AIDS in a patriarchal society amongst women/AGYW in Sub Saharan Africa and Zimbabwe?**

1. How does patriarchal society constrain AGYW living with or affected by HIV?
2. What are the methodological approaches underpinning research in AGYW’s experiences of HIV?
3. How do AGYW demonstrate capacity for agency and resistance in response to living with HIV (or being affected by HIV)?

Abbreviations

ALHIV-adolescents living with HIV

ART-Antiretroviral therapy

PMTCT-prevention of mother-To-Child Transmission

SRH- Sexual and reproductive health

**Sub-theme 1: Psychosocial distress**

**Codes:**

Presence of social support reduces distress associated with ART nonadherence (1)

The more distressed the more likely it is to miss pills & ART (1)

ALHIV experiencing psychosocial problems reduce ART adherence (8)

Activities of daily living, major negative events & quality of life are stressors that cause distress (12)

Religiosity & Pentecostalism was associated with psychological distress (12)

ALHIV experienced reported low levels of fatigue, pain, insomnia & distress (35)

Psychosocial challenges experienced by perinatally AGLHIV are stigma & discrimination, status disclosure, managing relationships & ART adherence (25)

AGLHIV experienced psychosocial challenges including lack of supervision, poor mental & physical health (25)

**Sub-theme 2: Antiretroviral therapy & nonadherence**

**Codes:**

Lack of private space, poor family/healthcare relationship & the size, taste & side effect affect ART adherence (8)

Negative psychosocial factors (lack of hope, low self-esteem, HIV identity, suicidal ideation) were associated with ART nonadherence (14)

Lack of food reduced ART adherence (5)

Lack of private space at school, dysfunctional family support & constant change of residence & multiple ART therapy were barriers to ART adherence (19)

Stigma, living conditions, lack of support for ALHIV prevented ART adherence (23)

Quest to normal (not taking cART) caused ART nonadherence (23)

ALHIV missed ART & clinic appointments due to multiple barriers (30)

ALHIV reported missing doses of ART (due to stigma, forgetfulness, travel away from home, being busy & being overwhelmed) (32)

**Sub-theme 3: Stigma & discrimination**

**Codes:**

AGYW experience stigma & discrimination within their social context (2)

Positive social capital enabled them to cope with this (2)

ALHIV experienced recurrent stigma & discrimination (19)

ALHIV were stigmatised & discriminated in their local communities (21)

ALHIV who experienced stigma & discrimination experience psychosocial challenges at school (27)

ALHIV experienced internalised stigma (30)

**Sub-theme 4: Exercising agency & resilience**

**Codes:**

ALYW LHIV are agentic and resilient driven by peer support & local cultural beliefs (3)

ALHIV experienced peer, relative support, healthcare support & psychological support interventions (14)

Young people demonstrated agency to be tested for HIV (19)

ALHIV are optimistic & determined to keep control of their lives (29)

Despite the burden of caring for the sick the child-carers demonstrated endurance, resilience & fortitude (40)

Some young carers drew inspiration to care for HIV relatives from cultural & social cohesion (16)

**Sub-theme 5: Negotiating HIV status disclosure**

**Codes:**

Fear of unsanctioned disclosure & stigma prevented ALHIV from sharing experiences of LHIV (5)

Two different outcomes of disclosure (negative vs positive reaction) (15)

Barriers of HIV disclosure local cultural norm, fear of stigma & rejection & wrong perception that children will not understand (17)

Outcomes of disclosure negative (anxiety, depression feeling guilty) vs interpersonal (access to ART support) & psychosocial support that complicated their lives (17)

Illness preceded disclosure & ART (19

HIV disclosure has two outcomes for ALHIV 1 abuse & abandonment 2 support, love & access to treatment & healthcare service (3)

Perinatally HIV infected ado. Preferred to have disclosure taking place in clinic settings (18)

Recurrent stigma & discrimination prevented disclosure in ALHIV (10)

Higher % of ALHIV experienced better family support than peer support post disclosure (10)

Young girls delayed disclosing to family members (21)

ALHIV worried about disclosing to sexual partners (24)

ALHIV delayed access to services & treatment (21)

**Sub-theme 6: Lack of comprehensive HIV knowledge**

**Codes:**

Inaccurate information about HIV by parents/guardians delayed HIV diagnosis & access to HIV care (29)

ALHIV had restricted access to accurate HIV knowledge (24)

Some participants showed lack of comprehensive HIV knowledge (5)

ALHIV have poor comprehensive HIV & SRH knowledge (29)

**Sub-theme 7: Peer mentoring & networking**

**Codes:**

ALHIV yearned for peer networking with other ALHIV (5)

Peer support from ALHIV created supportive network that enabled ART adherence (19)

Support group provided social & vocational skills (29)

Peer shared experiences by ALHIV facilitated sharing of age specific HIV information (18)

AGYW mothers preferred peer led PMTCT mentors because they were non-judgemental (9)

ART clinics were safe havens for ALHIV (they meet peers & talked about HIV without fear of disclosure & stigma)

ALHIV developed strong community social bond with peers (18)

**Sub-theme 8: Psychological stressors**

**Codes:**

ALHIV experienced more stressors because of their activities of daily living (frequent hospital appointments, ART regime, negotiating adolescence, stigma & failure to access social support) (11)

Psychological stressors lead to dysfunctional behaviour (risky sexual behaviour, ART nonadherence & mental health issues) (11)

**Sub-theme 9: absence of permanent residence**

**Codes:**

Frequent residential relocation of ALHIV resulted in late teen status disclosure (19)

Lack of family support (20)

Constant change of residence (20)

**Sub-theme 10: Coping with HIV status**

**Codes:**

Perinatally LHIV coped better with HIV identity vs heterosexually infected who felt guilty failed to cope with disclosure (19)

ALHIV had no one to talk to about their illness due to fear of unintended disclose & stigma (5)

**Sub-theme 11: Unresponsive school system**

**Codes:**

School environments lack a system responsive to specific ALHIV’s needs (27)

**Sub-theme 12: supportive psychosocial resources**

**Codes:**

Presence of social services facilitate ART adherence & acceptance of diagnosis (29)

Coping strategies for ALHIV are ART adherence, limited disclosure, social support & treatment optimism (25)

**Sub-theme 13: Inadequate Sexual & Reproductive Health (SRH)**

**Codes:**

SRH is underdeveloped to deal with sexual needs of ALHIV (29)

HIV & sexual & reproductive health failed to meet the needs of ALHIV in Zimbabwe (3)

ALHIV had lack of SRH, guidance & unmet sexual health needs (24)

ALHIV reported lack contraceptive counselling & multiple pregnancies (21)

**Sub-theme 14: Unsuitable living conditions**

**Codes:**

Witnessing violence at home; use of alcohol & poor self-efficacy were strongly associated with ART non-adherence & missing clinic appointments (32)

Overcrowded households & regimental school environment caused ART non adherence (23)

**Sub-theme 15: Relying on best friends**

**Codes:**

Best friend provided belief and strength (29)

Talking to a best friend about HIV diagnosis helped (29)

**Sub-theme 16: strained interpersonal relationships**

**Codes:**

Delay to disclose ALHIV’s status & misinformation created resentment in ALHIV (29)

ALHIV resented guardians/parents silence (19)

ALHIV experienced ill treatment from guardians because of their HIV status (19)

**Sub-theme 17: Constrained sexual partnership development**

**Codes:**

ALHIV delayed disclosing their HIV status to sexual partners (29)

ALHIV failed to express their sexuality caused by fear of the complexities (misconceptions of harmful sex in the context of HIV) of having sexual relationships while LHIV (24)

ALHIV rationalised sexual abstinence indefinitely (24)

ALHIV debuted sex earlier than males (37)

Taking ART; being popular & LHIV was a risk factor for earlier sex debut for ALHIV (37)

**Sub-theme 18: Inappropriate western developed tools & interventions**

**Code:**

Western constructed assessment tools were not culturally appropriate for the Tswana ALHIV (36)

There is lack of age-appropriate tools for communicating HIV parent’s HIV status or ALHIV (29)

HIV disclosure guidelines not applicable to Sub Saharan Africa setting (15)

**Sub-theme 19: HIV diagnosis & denial**

**Code:**

The burden of HIV diagnosis can spiral out of control if poor managed (29)

Some ALHIV were found to be in denial (3)

Young girls reported loneliness, fear & denial upon receiving HIV results (21)

**Sub-theme 20: local culture & ALHIV’s autonomy**

**Code:**

Shona cultural norms takes away ALHIV’s autonomy to control fertility by pressuring them to bear children (3)

**Sub-theme 21: Poor psychosocial adjustment**

**Codes:**

Exposure to HIV caused poorer psychosocial adjustment compared to children/orphans not exposed (4)

Children of parents LHIV or orphaned by HIV experienced higher conduct problems, peer problems & mental health issues (4)

Children orphaned by HIV experienced symbols of death, reminiscing about life experiences with departed parents & did not disclose cause of death of parents (13)

Children orphaned by HIV experienced conduct & behavioural challenges with their peers (39)

**Sub-theme 22: Lack of age specific health-care centres**

**Codes:**

HIV testing centres are not user friendly for youths, schools maybe a better option (6)

Minors receiving HIV results alone (21)

**Sub-theme 23: stunted development**

**Codes:**

ALHIV experienced higher health abnormalities; lower quality of life & wellbeing (7)

ALHIV reported higher health & education needs; stunted development; cognitive challenges; absenteeism & frequent clinic appointments (6)

**Sub-theme 24: Socioeconomic factors**

**Codes:**

ALHIV and pregnant mothers experienced ART nonadherence & PMTCT due to poverty caused by limited access to means of production (9)

AMLHIV experienced economic disempowerment & financial dependence & lack of financial support (9)

Poverty caused lack of food & stifled transition into adulthood (20)

Lack of food affected ART adherence (5)

Poverty ALHIV to experience psychosocial distress (27)

Lack of food to counteract ART side effects (9)

**Sub-theme 25: Caring for chronically ill HIV relatives**

**Codes:**

Children coped with caring responsibilities despite challenging circumstances (16)

Some young carers viewed the task as damaging to their lives (16)

**Sub-theme 26: Limited social support**

**Codes:**

Inadequate family & partner support leads to ↓ ART & PMTCT uptake in ALHIV

ALHIV experienced lack of counselling before & post HIV testing (21)

No support for children caring for HIV terminally ill before and post death (40)

**Sub-theme 27: experiencing fatigue**

**Code:**

ALHIV reported increased level of fatigue associated with sleep deprivation & mood swings (26)

**Sub-theme 28: Mental health issues**

**Codes:**

63% of ALHIV experienced depression & psychosocial wellbeing was poor (21)

ALHIV experienced depression & suicidal ideation associated with HIV diagnosis, loss of family member & bulling due to ART (31)

ALHIV develop mental health issue associated with bullying & stigma leading to suicide ideation (30)

**Sub-theme 29: dysfunctional behaviour**

**Code:**

Being pregnant and ALHIV placed young women at higher risk of alcohol use, mental health challenges & depression (33)

**Sub-theme 30: High risk sexual behaviours**

**Codes:**

ALHIV was a risk factor for not using contraceptives & condoms (38)

ALHIV were not likely to initiate contraception (38)

Being married or relationship increased the likelihood (38)

**Sub-theme 31: Retain self-efficacy**

**Codes:**

Adolescent mothers LHIV demonstrated belief and motivation to keep the infant’s health (9)

**Sub-theme 32: Safe environment**

**Codes:**

Schooling is a protective assert for ALHIV (37)

**Sub-theme 33: Personal aspirations**

**Codes:**

Adolescent Mothers LHIV wanted facilities to address stigma, poverty, food security & their complex health care needs (9)

**Sub-theme 34: Fear of rejection and isolation**

**Codes:**

The burden of caring & stigma isolated young carers & prevented them from sharing their emotions (40)

A feeling of isolation and rejection both self-imposed & anticipated for ALHIV (14)

**Sub-theme 35: Spirituality/religion seeking**

**Codes:**

Frequency of seeking religious services increases the likelihood that they will not adhere to ART (1)

Religious institutions have stigmatising attitudes & beliefs (1)

ALHIV experienced discrimination within the church (19)

Religiosity can endorse the external locus of control (god will cure me) (1)

**Sub-theme 36: HIV testing**

**Codes:**

Guardians/parents delayed to get children HIV tested (19)

HIV testing & treatment preceded multiple illness (19)

Minors unaccompanied when diagnosed of HIV (2

**Sub-theme 37: Family planning**

**Code**:

Lack of contraceptive counselling & unwanted pregnancies (21)

**Sub-theme 38: Coping with HIV related everyday life experiences**

**Codes:**

Activities of daily living & HIV related quality of live (12)

Negative major events (12)

Maintaining secrecy of HIV cause of death (13)

Burden of taking multiple ART daily (21)

Burden of HIV identity & sense of isolation & rejection; low self-esteem & hope (14)

Lack of protection (14)

Experiences of grief (14)

**Sub-theme 39 wider community environment**

**Codes:**

Failure to access care post HIV diagnosis (21)

Dropping out of care (21)

**Sub-themes and overarching themes**

| **Overarch-ing themes** | **Relational & psychosocial challenges** | **Managing health in everyday life** | **Inhibiting sexual expression** | **Agency and resilience** | **Poverty, stigma & discrimination** | **Personal space & social support** |
| --- | --- | --- | --- | --- | --- | --- |
| **Sub-themes** | -Psychological stressors  -Psychosocial distress  -Fear of rejection and isolation  -Mental health issues  -Dysfunctional behaviour  Depression | -Pentecostalism & religiosity  -HIV testing  -HIV diagnosis & denial  -Delayed disclosure  -Negotiating HIV disclosure  -Coping with HIV everyday life experiences  -Delayed disclosure  Antiretroviral therapy | -Lack of comprehensive HIV knowledge  -Inadequate sexual and reproductive health service  -Constrained sexual partnership development  -High risk sexual behaviours  -Strained interpersonal relationships  -Counselling  -Inadequate family planning | -Peer mentoring and networking  -Retaining self-efficacy  -Expressing agency and resilience  -Personal aspirations  -Safe environment  -Supportive psychosocial resources | -Socioeconomic challenges  -Caring for chronically HIV bedridden  -Stigma and discrimination  -Local culture versus personal autonomy  -Negative experience for ALHIV from poor socio context  -Delayed HIV disclosure | -Stunted development  -Absence of permanent residence  -Limited social support  -Unresponsive school system  -Lack of age specific health-care centre  -Poor background & unsuitable living conditions  -Relying on best friends  -Enlisting family members  -Spirituality and religiosity |
