# Supplementary material for: Adolescent Girls and Young Women’s Experiences of Living with HIV in the Context of Patriarchal Culture in Sub-Saharan Africa: A Scoping Review
Source: AIDS Behav. 2022 Nov 1;27(5):1365–79. doi: 10.1007/s10461-022-03872-6 (PMC10129999; doi:10.1007/s10461-022-03872-6)
Supplement: Supplementary file 5 — Supplementary Material 5 [file 10461_2022_3872_MOESM5_ESM.docx]

**Table 1: Inclusion and exclusion criteria of the included studies**

| **Inclusion**   - Primary studies and unpublished grey literature - Any study design addressing the experiences of living with (or being affected by) HIV in AGYW aged 15 to 24 years - Studies with at least a third of the participants who are AGYW living with (or affected by) HIV - Studies published in English with full text - Time limited from 1983 to 2021 - Studies on participants living in sub-Saharan Africa   **Exclusion**   - Studies not published in English - Secondary studies - Studies of AGYW living in countries outside sub-Saharan Africa - studies of other populations living with HIV not AGYW aged 15 to 24 years old. |
| --- |
