# Supplementary material for: Adolescent Girls and Young Women’s Experiences of Living with HIV in the Context of Patriarchal Culture in Sub-Saharan Africa: A Scoping Review
Source: AIDS Behav. 2022 Nov 1;27(5):1365–79. doi: 10.1007/s10461-022-03872-6 (PMC10129999; doi:10.1007/s10461-022-03872-6)
Supplement: Supplementary file 6 — Supplementary Material 6 [file 10461_2022_3872_MOESM6_ESM.docx]

| **Authors; year &**  **Table 2: Data extraction chart showing a summary to describe study characteristics & results of AGYW’s experiences of HIV n = 40**  **Country** | **-Aims/objectives**  **-Research Question(s)** | **Methodology & sample** | **Key findings & outcomes** | **Gap in knowledge** | **Comments** |
| --- | --- | --- | --- | --- | --- |
| Mutumba et al., 2016  Uganda | **Aim:** Examining the relationship between psychological distress & ART adherence, & effects of psychological resources on ART adherence | Cross sectional survey  -464 (F 249/53.4%) AGYW aged 12-19 years old | -Spirituality, religiosity, social support, coping strategies & optimism & satisfaction social support reduce stress & support ART adherence  -Psychosocial distress & frequency of seeking religious was associated with grater odds of missing pills & non-adherence to ART | Limited studies conducted investigating psychosocial resources & mental health impact on ART adherence in SSA  -AGYW bystanders in the research process | The finding “frequency of praying & attending religious services increased non-adherence” disputed earlier finding associating religious seeking behaviour with positive health outcomes in adults PLWH |
| Adegoke & Steyn 2011  Nigeria | **Aim**: explore how the HIV +ve AGYW were able to mitigate negative effects of their experiences of HIV infection & experience resilience through photo voice technique | Participatory Action Research: using photo-voice technique & narratives  -5 girls aged14-20 yrs | -Stigma & discrimination affected AGYW LHIV’s wellbeing  - AGYW LHIV used social capital to be agentic & resilient to sustain wellbeing & help other HIV +ve young people  - derived from the local cultural beliefs of placing high value on hard work, education, marriage, dressing & religion | -There is dearth of research on resilience among Yoruba AGYW living with HIV | -The challenge of Sub-Saharan Africa Culture which viewed youth sexuality as a problem not a positive part of youth development is persistent |
| Zamudio-Haas et al., 2012  Zimbabwe | **Aim:** Explore how young women living with HIV negotiate disclosure and how it impacts on partnership formation, decisions for family planning & desire raising children | Qualitative study.  28 young women aged between 16-20 | - Shona cultural norms pressure young women (YW) to bear multiple children  -HIV disclosure to partners is stressful experience for YW associated with abuse & abandonment or support & love  -HIV & sexual & reproductive health services (HSRHS) inadequate in Zimbabwe: -ALHIV desired to raise a family & children | There is no mechanisms or policy to help YW negotiate disclosure beyond the instruction inform their partners; -AGYW are bystanders in the research process | -25% of the sample chose not disclose HIV status to partners |
| Doku, P. N. 2010  Ghana | **Aim**: Gain a better understanding of how orphaned children or living with parents living with HIV are affected by the HIV pandemic | Quantitative design using questionnaires 149 in preadolescents 10-14 & post adolescents 15-19 | -Orphans & children exposed to parents infected by HIV have poorer psychosocial adjustment like higher symptoms of conduct problems, peer problems & depression & anxiety compared to those without the HIV experiences | Further research is needed on HIV orphaned children experience HIV  -The research is non-participatory | Findings from this research differed to earlier results finding no significant difference between age in all assessed symptoms |
| Enimil et al., 2016  Ghana | **Aim**: To understand experiences of ADLHIV in Ghana to identify ways to improve HIV engagement & quality of life | Mixed method: 40 adolescents living with HIV (ALHIV) aged 12-19 | -Lower quality of life psychological, wellbeing and social relationships; -Lack of comprehensive HIV knowledge Food insecurity hampered ART adherence  -Fear of unsanctioned disclosure and stigma limited disclosure | Lack of research on the experiences of adolescents living with HIV & AGYW as active participants | Results identified an unexpected barrier to ART adherence of food security as ART is taken with food |
| Toska et al., 2019  South Africa | **Aim:** Examine the differences in educational outcomes for ALHIV to identify educational markers for targeting HIV testing, counselling & linkages to care | Quantitative study  1063 ALHIV & 456 HIV –ve  (Female 57%) | -ALHIV reported higher rates of health issues, accessing school feeding schemes, educational delay, cognitive difficulties, maternally orphaned, being chronically sick, missing school to attend clinic & absenteeism  - AGYW prefer HIV youth user-friendly services | A participatory research methodology that focuses on the needs & experiences of harder to reach ALHIV in & out of schools is needed | There is a strong correlation: ALHIV was associated with worse physical & mental health, increased rates of chronic illness, cognitive difficulties, absenteeism & educational delay |
| Nkwata et al., 2017  Uganda | **Obj:** To examine quality of life in perinatally HIV-infected or HIV-exposed uninfected vs health HIV unexposed uninfected (HUU) children during school-age/adolescence | Retrospective cohort study  168 school aged children 6-18  Female 46% | -PHIV & had higher healthy abnormality, lower QOL, wellbeing, vigour/vitality score compared to PHEU or HUU | There is dearth of participatory research the personal and relational experiences of HIV ALHIV or HIV exposed AGYW |  |
| Okawa et al., 2018  Zambia | **Aim**: Examine adolescents’ depressive symptoms & ART adherence | Mixed method study  200 adolescents aged 15-19  Female 57% | -ALHIV had psychosocial distress & little HIV knowledge causing ↓ART adherence; -Loss of parents especially mother triggers psychosocial distress in AGYWLHIV  -Lack of space to take ART if not at home; - Poor interrelationships & size, taste & side effects of ART adherence | Few studies assessed the impact of physical & psychosocial changes on HAART adherence in AGYW using a participatory methodology | Late ALHIV spent more time away from home, need for customising medication schedule  -There is need for continuous education about HIV care & ART adherence |
| Carbone et al., 2019  Malawi | **Aim** Examine barriers to & facilitators of prevention of mothers-to-child transmission of HIV re for HIV infected AGYW in Malawi | Qualitative design  16 focus groups of 72 HIV infected adolescent mothers aged 15-19 | -Adolescent mothers living with HIV (AMLHIV) prefer peer-led no-judgemental PMCTCT support linking communities & facilities to systematically address barriers of stigma, poverty, health system complex & food security.  - structural barriers & poverty caused ART & PMTCT non- compliance; - Comprehensive gender empowerment strategies will promote PMCTC uptake | The is dearth of participatory research on how AGYW PMTCT programming can be integrated with socioeconomic interventions e.g cash transfers, income empowering activities | PMTCT be do more if tailored to the unique needs AGYW LHIV in SSA  e.g. need to be mentored by mentors LHIV |
| Nostlinger et al., 2015  Uganda & Kenya | **Aim**. Examine social HIV stigma, self-esteem, and social support among ALHIV | Quantitative study:  582 ALHIV 13-18; 263 boys (45.2%) & 319 girls (54.3%) | -Recurrent stigma prevented HIV disclosure  -Disclosure to peers is influenced by being older, an orphan, contributing to family income, regular visit to HIV clinic & better support from peers & HCs | Scarce research on factors influencing pre& post disclosure in ALHIV using participatory research | ALHIV need value confidence and safe environment to practice disclosing |
| Folayan et al., 2016 Nigeria | **Aim:** Explore the main sources of mental stress & describe the coping strategies used, if any, to mitigate stress by LHIV in Nigeria | Cross-sect. surv: sec. analysis- data limited to 600 (449/74.8% LHIV) ado. 10-19 yrs tested for HIV (girls 315/52.5%)  Total sample 1574 | -ALHIV had an increased odds stressor due to visiting the hospital regularly, adhering to ART, disclosing status, choosing partners & practising safe sex, LHIV, stigma, discrimination, sexual abuse and abandonment causing dysfunctional behaviour like substance use, risky sexual behaviours, non-adherence to ART and mental health issues | There is no participatory research on the source of mental stress for African ALHIV and the coping strategies they adopt | Psychosocial challenges & coping strategies used ALHIV are mitigated by cultural context they live in therefore its important individualise HIV care according to location |
| Mutumba et al., 2017 Uganda | **Purpose**: To expand understanding of the risk & protective factors for psychological distress among ALHIV in Uganda | Cross-sectional study  464 ALHIV aged 12-19 with 53.4%/249 females | -Stressors related to psychological distress for ALHIV were ADLs, major negative events, HIV related quality of life & stigma  -Protective factors were psychosocial resources | There is no participatory research on the psychological distress & its impact among ALHIV in sub-Saharan Africa | Religious coping is a strategy for ALHIV in SSH as many ALHIV are religious |
| Thupayagale-Tshweneagae and Benedict 2011 Botswana | **Purpose:** To describe the burden of silence borne by adolescents orphaned by HIV | Phenomenological research: using photo-voices  Aged 14-18 years old | -Symbols of death like constant reminders of dead parents & relatives was shown by orphans reminiscing about what they used to do with their parents  -maintaining the silence of cause of death was a constant stressor | There is dearth of research using photo-voices on ALHV or affected by HIV | Using peer support of other adolescents orphaned by HIV as in mother2mentor model |
| Willis et al., 2018  Zimbabwe | **Aim:** To explore the experience & manifestations of depression in ALHIV in Zimbabwe to inform development intervention | Qualitative research using Photo-voices in 21 ALHIV  Aged 15-19 (Female 10/48%) | -AGYW LHIV experienced being different, longing for recognition, sense of isolation & rejection, low self-esteem, lack of protection, grief & loss, worry, pain, stress, lack of hope and suicidal ideation (slow suicide by ART non-adherence) | There is limited research on the prevalence, occurrence & management of depression in ALHIV & non using participatory research | The vicarious impact of family & peers causing or mitigating depression in ALHIV |
| Mutumba et al., 2015  Uganda | **Purpose:** To explore disclosure experiences of HIV infected adolescent in Uganda | Qualitative study  13 ALHIV aged 13-19 years  Females 20/53% | - Preparation for disclosure: misleading information given to ado -Response to disclosure: feeling bad; isolated, disbelief, fear, worry, anger hopelessness, bitterness & suicide ideation -Positive reaction: relief about knowing status  -Lack of support post disclosure | -There is no participatory research must focus on AGYW’s disclosure preferences & how they experience HIV disclosure | Disclosure guidelines are not applicable to sub Saharan African as disclosure is done after adolescence |
| Skovdal and Ogutu 2009  Kenya | **Aim**: To explore the psychosocial well-being of children providing care for people chronically ill from AIDS | Qualitative research: Action research project  48 young carers aged 11 to 17 | -Most children naturally got on with the challenges of providing care  -The meanings YC attach to their circumstances is based on the social environment with local culture  -a minority of children viewed caring either as a relief or something which has caused damage to their lives | There is dearth of research on the psychosocial needs of ADO carers in the context of SSA from a participatory methodology perspective | ADO attached negative feelings to experiences as a result of the poverty endured but also identify benefits from caring experiences |
| Mburu et al., (2014) Zambia | **Research Questions:**  -what barriers to disclosure were encountered once the ado knows their HIV status.  -what factors influences their disclosure to others | Qualitative study  58 (interviews) adolescents aged 10-18 (28/50% female)  Focus groups 53 females 35/66% | -Local norms & fear of HIV stigma prevent open discussion of chn’s sexuality needs  -A presumption that adolescents would not understand HIV diagnosis; Barrier to disclosure to sexual partners: fear of rejection -Two outcomes of disclosure  -Personal; anxiety, depression, feeling guilty; Interpersonal; facilitated access to adherence support, psychosocial support but strained ado’s sexual relationships | Research on potential of trusted family members in enabling HIV disclosure process | HIV disclosure is influenced by cultural and interpersonal factors  -Disclosure in SSA is often a once only event not a process, ado. HIV status is often disclosed to 3^rd^ parties without their consent |
| Kidia et al., 2014  Zimbabwe | **Aim:** To understand how perinatally-infected ado learn about their HIV status & examine their preferences for the disclosure processes | Qualitative study  31 (13 male 17 female/54%) perinantally-infected ado. aged 16-20 | HCW encouraged caregivers to initiate disclosure to younger chn in their homes  -ado preferred disclosure to take place in the presence of HCW at a clinic setting  -Peer shared experiences facilitated learning of specific aged, related HIV information about living with HIV | Current WHO HIV disclosure guidelines in ADO are not specific to sub–Saharan African AGYW | HIV disclosure carried out in a clinical setting overcome barriers of HIV disclosure  -Given that the majority of ALHIV live in SSA, research is required to investigate the influencing specific sociocultural context |
| Sui et al., 2016 Uganda | **Aim:** Exploring young people (YP)’s trajectories towards HIV treatment: how, when, and with whom testing, and treatment decisions are made & the role of ado. themselves in this process | Qualitative study.  20 Young people aged 15-23 (10 females/50%) on ART | -Mobile residence caused delayed disclosure & HIV testing & treatment preceded illness; YP reported guardian ill treatment, lack of resources for edu and income & resented parental/carer silence  -YP’s had personal agency to be tested  Recurrent stigma & discrimination  - heterosexually acquired HIV YP felt guilt, regret & struggled with disclosure; - Counselling helped adherence & coping | There is a dearth of research how YP exposed to HIV access testing & subsequent services from a participatory methodology point of view | YP described how their own agency prompted testing and treatment |
| MacCarthy et al., 2018 Uganda | **Aim:** To understand barriers to ART adherence specific to young people aged 14-24 | Qualitative research  25 YP aged 14-24 (F 11/44%) | -Poverty caused lack of food & independence transition into adulthood  -lack of private space caused ART non-adherence; -inconsistent & unreliable family support & constant change of residence; Peer support creates supportive network to ART adherence  -burden of taking multiple drugs led to “drug holiday” | There is dearth of YP specific barriers to ART adherence | Adolescent & youth specific challenges of disclosure & coping with HIV were consistent in all barriers |
| Luseno et al., 2019  Kenya | **Aim:** To explore the challenges in promoting the health of ALHIV/parenting and preventing onward transmission | Qualitative research  28 ALHIV aged 15-19 (Female 21/75%); 15 HIV providers | -Social support critical during HIV testing & adherence to ART & PMTCT programmes  -ALHIV reported lack of support during HIV testing, experiences of fear & denial & delay in disclosure and accessing treatment  -Lack of contraceptive counselling & unwanted pregnancies | There is a dearth of research in pregnant ALHIV or parenting  -The methodology used is not participatory | Inadequate HIV services negatively impact ado. engagement HIV treatment before sexual debut & conceiving; in PMTCT & after pregnancy |
| Mavhu et al., 2013  Zimbabwe | **Objective:** To strengthen evidence base for psychosocial interventions to support YP LHIV in Southern Africa | Mixed method approach  Young people YP aged 15-18: 229/310 (74%)/59% females | -self-reported ART adherence was sub-optimal  -Psychosocial well-being was poor  -63% were at risk of depression  -HIV +ve YP faced verbal abuse, stigma & discrimination challenges | Scarce research on the interventions addressing the psychosocial needs, transition to adulthood for HIV positive young people | YP LHIV face stunted growth, recurrent illness & admission poor school attendance, delayed puberty, skin disease & intellectual impairment |
| Mutwa et al., 2013  Rwanda | **Objective:**  To better understand cART adherence barriers & success in adolescents in Rwanda | Qualitative study  42 peri-natally ALHIV aged 12-21 (female 45%) | Three themes hampered cART adherence  -Stigma (perceived & experienced); inadvertent HIV disclosure & living conditions (orphanage, foster care &boarding schools)  -Quest to be normal (not taking cART, forced to disclose & stigmatized) | There is lack of participatory research exploring the experiences of ALHIV & the impact of cART on their lives | ALHIV need space to take cART in privacy.  ALHIV desire to be normal if not understood or supported leads non-adherence |
| Busza et al., 2013  Tanzania | **Aim:** To better understand the needs & experiences of ALHIV in the context of home-based care programme to improve existing services | Qualitative study  14 (Female 5/33%) ALHIV aged 15-19  12 primary caregivers | -Sexuality induced anxiety in ALHIV-fear of complications caused by & relationships  -fear of disclosing to partners, their health & risk of infection partners  -Rationalised abstinence indefinitely | There is a gap in ALHIV’s perceptions of sexuality, ability to access SHR services & interpretation of HIV prevention messages | ALHIV have inadequate access to & guidance to comprehensive HIV knowledge resulting in unmet sexual health needs |
| Mutumba et al., 2015  Uganda | **Objective**: To identify the psychosocial challenges & coping strategies among perinatal HIV infected adolescents in Uganda | Qualitative study  38 (53%/20) ALHIV aged 12-19 | -Psychosocial challenges: discrimination, stigma & disclosure & ART  -Coping strategies were ART adherence, limited disclosure, treatment optimism, social support, rationalization, social comparison, spirituality/religiosity, avoidance & distraction | There is dearth of research on how patriarchal norms influence psychosocial factors that affect ALHIV in SSA & they cope with these challenges | ALHIV face psychosocial problems caused by the complexities of negotiating adolescence within the context of a stigmatised & potentially fatal illness |
| Coetzee et al., 2019  South Africa | **Objective**: Examine the correlation between fatigue & demographic & psychosocial variables | Quantitative research  134 ALHIV aged 11-18  (Females 78/58%) | -A quarter of ALHIV reported increased levels of fatigue associated with poor sleeping & mood swings  -age, depression & sleeping problems caused variance in fatigue | Further research on the notion of fatigue ALHIV is needed | Fatigue can be a subtle yet disabling challenge in ALHIV |
| Abubakar et al., 2016  Kenya | **Objective:** Investigate psychosocial challenges faced by ALHIV in school system within SSA context | Qualitative research  19 ado aged 12-17 (Females 8/47% and 25 key informants | -Poverty, poor mental & physical health, the lack of a school system responsive to their specific needs, disclosure, recurrent stigma, ART adherence & lack of close supervision | There is limited research on the lived experiences and challenges faced by ALHIV in a school system in the SSA/patriarchal context | ALHIV’s outcomes are affected by a complex of social, economic and medical challenges |
| Kemigisha et al., 2019  Uganda | **Aim:** To determine the prevalence of depressive symptoms & their associated factors among ALHIV in Uganda | Cross sectional survey  336 ALHIV aged 10-19 (Female 209/62%) | -46% had depressive symptoms  -odds of depression was higher in ALHIV older than 15 years, had disclosed & travelling to clinics to collect ART | There is limited research on mental health issues in ALHIV specific to SSA | ALHIV have mental health challenges due to stage of dev., social, medical & psychosocial stressors |
| Hodgson et al., 2012  Zambia | **AIM**: Explore & document informational, psychosocial, SRH needs of ALHIV aged 10-19 in Zambia & identify gaps between needs & existing services | Qualitative explorative study  111 (female 63/58%) ALHIV aged 10-19  59 key informants | -Social support + family increased ART adherence & diagnosis acceptance  -SRH for ALHIV are underdeveloped, sexuality & HIV discussions a taboo  -HIV services provided a safe haven  -stigma led to delayed or non-disclosure  HIV ART clinic safe haven, meet peers & receive support with fear of stigma | There is no research where AGYW co-designed age specific services for ALHIV in low & middle-income countries | LMIC have poor SRH & psychosocial intervention for ALHIV |
| Ashaba et al., 2018  Malawi | **Objective;** To estimate the association between internalized stigma, bullying, major depressive disorder (MDD) and suicidality | Cross-sectional study  224 (131/58%) ALHIV aged 13-17 | -16% of participants had MDD; 13% suicidality; 4% high risk suicidality; 41% internalised stigma; 43% reported bullying episodes  -MDD was associated with bullying with suicidality associated with bullying & stigma | There is limited research on the experiences of depression & suicide ideation in ALHIV in SSA | Depression is a fundamental issue in ALHIV as it is correlated to ART non-adherence & poor HIV outcomes |
| Kim et al., 2015 Malawi | **Aim**: To determine the associated between depression & HIV & use the knowledge to dev. & improve comprehensive holistic ado. ART care | Cross-sectional study  562 aged 12-18 ALHIV (Female 325/56.1%) | AGYWs reported depression & bullying  -Depression was associated with few yrs of schooling, death of family member, failing at school, having a sex partner, not disclosed, bullied due to ART | There is scarce research on potential correlation of depression & HIV infection among ALHIV in the context of SSA patriarchal norms | Examine SSA multifunctional aetiology of HIV & depression; there is a bidirectional relationship between HIV & depression each increasing the other |
| Kim et al., 2017 Malawi | **Aim**: To examine levels of levels of self-reported ART adherence, barriers to adherence & factors associated with non-adherence amongst ALHIV in Malawi | Cross-sectional study  519 AHIV aged 12-18 (female 290/56%) | -AGYW reported missing doses  -Barriers to ART adherence: forgetting travel from home, busy with activities, overwhelmed, feeling stigmatized outside home & inside home & other factors alcohol, missed clinic appoint, witnessing violence at home; poor self-efficacy | There is a dearth of research in ALHIV adherence to ART in Zimbabwean context | 40% of incidences of HIV occur in young adults aged 15-24 |
| Wong et al., 2016 South Africa | **Goal**: To describe demographic & psychosocial factors in HIV infected pregnant women, comparing YW 18-24 to ≥25 years | Observational cohort study  658 (160/34% aged 18-24 + 465 ≥25 years) | -Young pregnant women were at greater odds of increased risk alcohol related harm; suicidal ideation & self-harm & depressive symptoms | No participatory research-based strategies to address psychosocial factors affecting pregnant ALHIV in SSA patriarchal context | Understanding factors influencing behaviour of pregnant ALHIV has implications for their children & families |
| Petersen et al., 2010  South Africa | **Aim:** To understand psychosocial challenges & protective factors supporting socio-emotional coping in ALHV & caregivers to inform mental health & HIV strategies in SA | Qualitative study  25 ALHIV aged 14-16 (female 12/48%) & 15 caregivers | -ALHIV struggled with disclosure, the HIV+ identity; external stigma & discrimination & loosing parents  -ART, HIV information, future orientation & social support were reported to be key to promoting supportive caregiving | There are no strategies specifically designed with ALHIV for their specific psychosocial needs |  |
| Kagee et al., 2019 South Africa | **Aim:** examine the relationship between fatigues, sleep disturbance, depression. | Cross-sectional study  134 ALHIV aged 111-18 (females 78/58.2%) anxiety, pain & QOL among ALHIV on ART | -participants reported low levels of fatigue, insomnia, distress and pain and quality of life  Results are different from the commonly held belief that ALHIV from poor socio contexts would experience lower QoL | Further research is needed to explore AGYW’s agency to understand how AGYW cope with living with being affected by HIV | ALHIV must viewed as agentic not mere victims in the HIV research.  -HIV policy makers can draw on ALHIV agency to develop future HIV strategies |
| Lowenthal et al., 2014  Botswana | **Aim:** To identify culturally specific factors to establishing local construct validity for culturally adapted tools for measuring psychosocial outcomes | Qualitative study  34 (18/53%) aged 10-19 ALHIV | -Western constructed assessment tools poorly perceived Tswana cultural context  -Ado. suggested that other tools were unsuitable & needed adjustments to obtain construct validity in Botswana  .an unexpected theme was noted denial of being HIV+ | Further research is needed in ado. ambivalence, ART adherence & HIV+ denial | The threat of ado ART non-adherence & size of challenge of HIV prevalence among perinantally HIV+ requires qualitative understanding |
| Bakeera-Kitaka et al., 2019 Uganda & Kenya | **Aim:** Identify determinants independently associated with the onset sex debut among ALHIV | Cross-sectional data  580 ALHIV aged 13-17 (Female 317/55%) | -20% females debuted sex earlier than males 20%  -Taking ART & LHIV increases the odds of sexual debut & Schooling is protective to girls not boys -Being popular ↑ the risk of early sex debut in girls | There is participatory research on AGYW’s personal and relational experiences leading to sex debut in ALHIV SSA | AGYW use agentic transactional sex as a means to achieve economic independence & socially identity |
| Buyeza-Kashesya et al., 2011 Uganda | **Aim**: To explore contraceptive decision-making & practice of HIV- & HIV+ youth | Cohort study  501 HIV- & 276 ALHIV aged 15-24 years (female 578/74.5%) | -ALHIV were likely not to consistently use condoms & contraceptives  -being HIV+ ↓the odds of initiating contraceptives but being in a relationship or married ↑the odds | There is a dearth of research on sexual & reproductive health decision making in HIV- & HIV+ young people in SSA patriarchal context | Being HIV+ is known to come with recurrent stigma. There is need to find out the impact of this stigma of self-efficacy of young people |
| Doku 2009 Ghana | **Aim:** To examine the impact of parental HIV/AIDS status & death on the mental health of children in Ghana | Cross-sectional survey  200 children aged 10-19 (orphaned of AIDS 50; orphaned of other causes 51 children LHIV parents 48; non-orphans 48) | -Children orphaned by HIV/AIDS had ↑peer challenges  -all orphaned groups had conduct problems  --emotional problems were common in all groups except non orphaned  -Hyperactivity was low in all the groups | There is limited research on the psychosocial effects of HIV/AIDS on children in Zimbabwe | HIV care is mainly focused on prevention than psychosocial input for children affects by HIV/AIDS |
| Demmer & Rothschild 2011 South Africa | Aim: To understand the experiences of adolescents bereavement post death of a sibling | Qualitative descriptive study  11 children aged 13-19 (females 8/73%) | -there was no support for HIV/AIDS child-carers before & post death  -Stigma & the burden of caring an HIV/AIDS bedridden relative isolated & restricted the child-carers from sharing their emotions but they demonstrated endurance, resilience & fortitude | Further research is needed to understand the bereavement children of family members who die of HIV/AIDS in the SSA patriarchal context | HIV/AIDS child-carers have little or no support before & after death of a family member |
